# The associations of maternal and paternal obesity with latent patterns of offspring BMI development between 7-17 years of age: pooled analyses of cohorts born in 1958 and 2001 in the United Kingdom

**DOI:** 10.1038/s41366-022-01237-6

**Published:** 2022-11-10

**Authors:** William Johnson, Snehal M Pinto Pereira, Silvia Costa, Jenifer L Baker, Tom Norris

**Affiliations:** 1 School of Sport, Exercise and Health Sciences, Loughborough University, Loughborough, UK; 2 UCL Division of Surgery & Interventional Science, University College London, London, UK; 3 Center for Clinical Research and Prevention, Bispebjerg and Frederiksberg Hospital, The Capital Region, Copenhagen, Denmark

## Abstract

**Objective:**

We aimed to 1) describe how the UK obesity epidemic reflects a change over time in the proportion of the population demonstrating adverse latent patterns of BMI development and 2) investigate the potential roles of maternal and paternal BMI in this secular process.

**Methods:**

We used serial BMI data between 7-17 years of age from 13220 boys and 12711 girls. Half the sample was born in 1958 and half in 2001. Sex-specific growth mixture models were developed. The relationships of maternal and paternal BMI and weight status with class membership were estimated using the 3-step BCH approach, with covariate adjustment.

**Results:**

The selected models had five classes. For each sex, in addition to the two largest normal weight classes, there were “normal weight increasing to overweight” (17% of boys and 20% of girls), “overweight increasing to obesity” (8% and 6%), and “overweight decreasing to normal weight” (3% and 6%) classes. More than 1-in-10 children from the 2001 birth cohort were in the “overweight increasing to obesity” class, compared to less than 1-in-30 from the 1958 birth cohort. Approximately 75% of the mothers and fathers of this class had overweight or obesity. When considered together, both maternal and paternal BMI were associated with latent class membership, with evidence of negative departure from additivity (i.e., the combined effect of maternal and paternal BMI was smaller than the sum of the individual effects). The odds of a girl belonging to the “overweight increasing to obesity” class (compared to the largest normal weight class) was 13.11 (8.74, 19.66) times higher if both parents had overweight or obesity (compared to both parents having normal weight); the equivalent estimate for boys was 9.01 (6.37, 12.75).

**Conclusions:**

The increase in obesity rates in the UK over more than 40 years has been partly driven by the growth of a sub-population demonstrating excess BMI gain during adolescence. Our results implicate both maternal and paternal BMI as correlates of this secular process.

## Introduction

The obesity epidemic is a major public health threat. According to the 2019/20 data from the National Child Measurement Programme in England, 10% of children in Reception (4-5 years) and 21% of children in Year 6 (10-11 years) had obesity.[1] Obesity rates then continue to increase into adulthood [2] due to the marked biological (e.g., decreased insulin sensitivity) and behavioural changes (e.g., decline in physical activity) that occur with puberty and during adolescence.[3] It is well known that the decade of life following the adiposity rebound at 5-7 years of age is a critical period in obesity development.[4] Few studies have, however, used growth mixture modelling to capture and describe the latent class (or classes) of children who share an average body mass index (BMI) trajectory that transitions from non-obese to obese during adolescence.[5–10]

Using data from the nationally representative United Kingdom (UK) birth cohort studies, we have previously shown a positive skewing of the BMI distribution at increasingly younger ages in more recently born cohorts.[11] Alternatively, the obesity epidemic can be viewed as a shift over time in the proportion of the population demonstrating obesogenic BMI trajectories. In a recent paper that applied growth mixture modelling to data from three UK birth cohorts, Norris et al revealed how the obesity epidemic partly reflects an increase in the proportion of the population belonging to a latent class characterised by an average BMI trajectory that started in the normal weight range at 11 years but ended in the overweight range at 42 years.[12] Compared to the 1946 birth year cohort, the 1970 cohort (but not the 1958 cohort) had a higher odds of belonging to this class rather than a referent class that was consistently normal weight. The same advanced approach has not been applied using the much more recently born 2001 cohort (in combination with an older cohort or cohorts) to understand secular changes in latent patterns of child-adolescent BMI development from before the obesity epidemic era to present day. Such an analysis would provide important information that cannot be provided by conventional growth curve modelling, an approach which has been widely used to describe cohort differences in mean BMI and obesity prevalence trajectories.[11, 13, 14] By viewing the evolution of the obesity epidemic in this traditional way, one fails to consider the possibility that higher/steeper mean BMI and obesity prevalence trajectories in more recently born cohorts could be the result of complex secular changes in the proportion of the population demonstrating different patterns of development. For example, the 2001 cohort would likely have a higher/steeper mean BMI trajectory than an older cohort, but this could be the result of a secular change towards more children having high-to-average BMI trajectories (e.g., 10% in 2001 cohort; 5% in older cohort), and average-to-high BMI trajectories (e.g., 30% in 2001 cohort; 5% in older cohort), and even consistently low BMI trajectories (e.g., 5% in 2001 cohort; 2.5% in older cohort).

Changes over time in rates of adulthood obesity have broadly mirrored those of childhood obesity, and parental obesity is arguably one the strongest determinants of offspring obesity.[15, 16] Previous analyses in the UK cohorts have demonstrated strong associations of both maternal and paternal BMI with offspring obesity that persisted well into adulthood and were not explained by traditional confounders or offspring lifestyle factors.[17–21] Such traditional analyses, however, impose linear constraints that are a simple representation of the complex way in which parental BMI might be related to offspring BMI trajectories. For example, a one-unit higher maternal BMI might be related to a 0.05 kg/m^2^/year steeper offspring BMI trajectory on average, but growth mixture modelling might provide additional information that higher maternal BMI is associated with greater odds of their child belonging to a small adolescent-onset of underweight group. A recent paper by Dos Santos et al found that maternal obesity was related to membership of unhealthy latent BMI trajectory classes (e.g., always obese) in the 2001 cohort,[22] and similar findings have been reported in other studies.[7, 8, 10, 23, 24] The majority of this literature, however, does not consider paternal BMI and inadvertently contributes to the “imbalance of DOHaD [development origins of health and disease] research towards the study of maternal pregnancy exposures”.[25, 26] The literature also largely comprises studies that have been conducted using data from one cohort of children born at one point in time, and the reported associations do not necessarily tell us anything about how maternal and/or paternal obesity might have contributed to the paediatric obesity epidemic.[27]

We aimed to 1) describe how the UK obesity epidemic reflects a change over time in the proportion of the population demonstrating adverse latent patterns of BMI development between 7-17 years of age and 2) investigate the potential roles of maternal and paternal BMI in this secular process.

## Methods

### Sample

The 1958 National Child Development Study (NCDS) is based on 17638 people born in one week in March 1958 in England, Scotland, and Wales; 920 immigrants born in the same week were incorporated during childhood.[28] The 2001 Millennium Cohort Study (MCS) is based on 18818 people born between September 2000 and January 2002 who were living in England, Scotland, Wales, or Northern Ireland at age 9 months.[29] Both studies have received ethical approval and obtained informed parental and/or participant consent; this information is available from the study websites and/or cohort profiles.

For inclusion in the present study, participants were required to have at least two values of BMI during the studied age range. The resulting sample comprised 13220 boys (49.1% from the 2001 MCS) and 12711 girls (50.1% from the 2001 MCS).

### Child BMI Data

In the 1958 NCDS, weight and height were measured at data collection sweeps at target ages of 7, 11, and 16 years. In the 2001 MCS, weight and height were measured at data collection sweeps at target ages of 7, 11, 14, and 17 years. All data were collected by trained individuals using similar instruments and measurement protocols. In total, there were 39184 BMI observations for boys (55.9% from the 2001 MCS) and 38144 for girls (57.1% from the 2001 MCS). Supplementary Tables 1-3 provide detailed description of the BMI data.

### Parental BMI data

In the 1958 NCDS, mothers’ weights were self-reported in 1969 (child aged 11 years) and heights were measured in 1958 (child aged 0 years), with missing height data supplemented from 1969 self-reports. Fathers’ weight and heights were both reported in 1969. While all the height data were recorded to the precision of one inch (2.54 cm), “weights were classified into one of 27 groups ranging from 6 stone 4 pounds (39.9 kg) to 19 stone 10 pounds (125.2 kg) in increments of 6 pounds (2.7 kg).”[19] Each parent was assigned a weight equal to the midpoint of their weight group. In the 2001 MCS, maternal and paternal body weights were self-reported, in stones & pounds or kilograms, at the 11-year sweep. Heights were also reported at the 11-year sweep, but the vast majority (95%) of these data were missing. Instead, we used maternal and paternal body heights self-reported, in feet & inches or centimetres, at the first (9-month) sweep. After converting all imperial data to metric, maternal and paternal BMIs were calculated as kg/m^2^. Weight status was defined as normal weight (<25.0 kg/m^2^), overweight (25.0–29.9 kg/m^2^), or obese (≥30.0 kg/m^2^).

### Covariates

In addition to birth cohort, offspring sex and ethnicity (white British vs other), and parental ages at the birth of the child, we considered three key measures of socioeconomic position. Tenure (own outright or mortgage vs other) was assessed at the 7-year sweep in the 1958 NCDS and at the 9-month sweep in the 2001 MCS. Father’s occupation was assessed at the 11-year sweep in both studies and was classified according to the Registrar General’s Social Class.[30] As in previous publications, for the 2001 MCS study only, we used 2618 observations of mother-figure occupational class when no father-figure was present in the household or when no valid father-figure occupational class data were available.[31–33] The age at which mothers left full time education was assessed at the 16-year sweep in the 1958 NCDS and at the 9-month sweep in the 2001 MCS.

In the 1958 NCDS, it was assumed that both mother and father were natural parents. In the 2001 MCS, the natural mother and natural father did the interviews in most cases. Nonetheless, we restricted all parental data in the 2001 MCS to that from natural parents.

### Statistical analyses

Descriptive statistics were produced, stratified by sex, for all children, 1958 NCDS children, and 2001 MCS children.

A separate growth mixture model for each sex was developed to identify distinct groups of individuals who had similar BMI trajectories between 7-17 years of age. The base model included a linear trajectory and default specifications in Mplus (i.e., variance and covariance terms, and the residual variance at each time point, freely estimated but constrained to be the same in each class), with the addition of T-scores to account for the fact that measurements were not taken at discrete ages (i.e., not all BMI values were taken at exactly 7, 11, 14, 16, or 17 years of age). Subsequently, model development considered a quadratic function for the trajectory shape, allowing the residual variances to differ across classes, and allowing the intercept variance to differ across classes. For each step of development, models with 1 to 6 class solutions were run. Supplementary Tables 4 & 5 show how the Bayesian information criterion (BIC) substantially improved with each step of model development. More complex and flexible models (e.g., allowing auto-correlated residuals) were tested but either did not converge or did not improve model fit.

To avoid convergence at local minima,[34] the final growth mixture models were fitted specifying up to 1000000 random starts (for 30 iterations), of which the best 200000 models (according to log-likelihood) were run to completion (STARTS = 1000000 200000; STITERATIONS = 30). A summary of the final mixture models, including measures of class separation, is presented in Supplementary Tables 6 & 7. Supplementary Figures 1a to 2f show the average fitted trajectories, superimposed on the International Obesity Task Force (IOTF) weight status ranges, for each model (i.e., boys or girls) and class solution (e.g., 1-6). The best class solutions were selected based on model fit (e.g., BIC), quality of classification or separation between the classes (e.g., entropy), and plausibility and interpretability of the average trajectories.

To investigate the relationship of auxiliary independent variables (e.g., birth cohort or parental BMI) with class membership we used the 3-step BCH (named after Bolck, Croon, & Hagenaars) approach in Mplus, using full information maximum likelihood (FIML) to handle missing data.[35] Briefly, this approach can be thought of as a multinomial logistic regression which appropriately accounts for the uncertainty in class membership. In addition to estimated odds ratios (OR), we obtained sample statistics (e.g., mean value of each independent variable in any given model) for each class, weighted by estimated class probabilities. All analyses were performed for each sex separately.

- Firstly, we ran models to estimate the relationships of birth cohort with class membership. We also did a crude analysis tabulating modal class membership against birth cohort.
- Secondly, we estimated the relationships of parental height (cm/10), BMI, and weight status with class membership, considering mothers and fathers, and each exposure, separately. Four sets of models were run: 1) unadjusted, 2) adjusted for birth cohort, 3) testing for effect modification by birth cohort (BMI exposures only), and 4) fully adjusted for maternal or paternal age, tenure, occupational class, maternal age left full-time education, and birth cohort.
- Thirdly, we considered maternal and paternal BMI, as well as their interaction, together in fully adjusted models. Because interactions in logistic models test departure from multiplicativity, we also computed and used the Relative Excess Risk due to Interaction (RERI) measure to test departure from additivity.[36]
- Fourthly, we tested a categorical exposure which considered maternal weight status and paternal weight status together (e.g., both parents had normal weight, mother (but not father) had overweight or obesity, father (but not mother) had overweight or obesity, both parents had overweight or obesity) in fully adjusted models. This analysis was also re-run with the following different groupings: both parents had normal weight, one parent had overweight, both parents had overweight, one or both parents had obesity. Supplementary Tables 8 & 9 describe how these exposures were computed.

As secondary analyses, we investigated the associations of each socioeconomic position variable with class membership, adjusting for birth cohort. In all models in this paper, occupational class and maternal education were entered as ridit scores for parsimony.[37] Presented estimates represent the contrast of the highest socioeconomic group (e.g., professional occupation) compared to the lowest (e.g., unskilled occupation).

## Results

Table 1 shows the descriptive statistics. When considering both cohorts combined in the total columns, the prevalence of parental obesity was approximately 11-13% in all instances, while overweight was more common in fathers than mothers (e.g., 40 vs 28%),

### Latent classes

**Figure 1:**
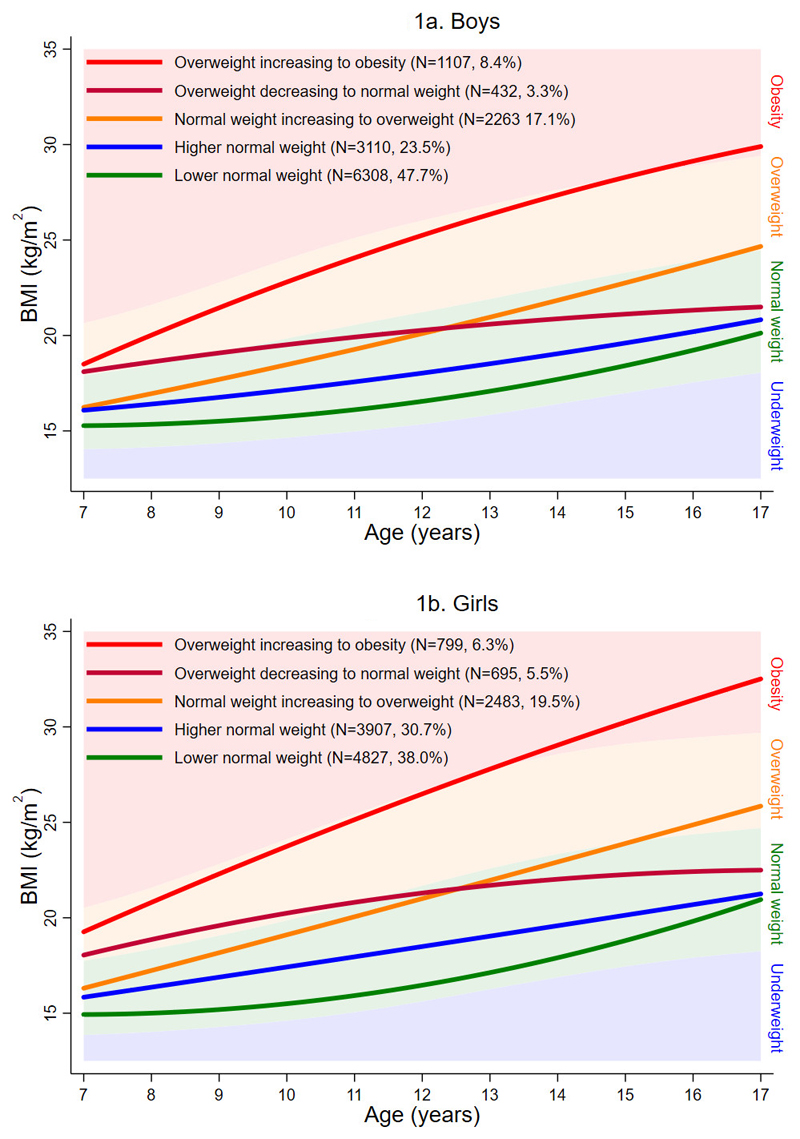
a. Average fitted trajectories from the final mixture model for boys, superimposed on the IOTF underweight, normal weight, overweight, and obesity ranges b. Average fitted trajectories from the final mixture model for girls, superimposed on the IOTF underweight, normal weight, overweight, and obesity ranges

For both boys and girls, a mixture model with five classes provided the best representation of the serial BMI data and the most plausible solution. Figure 1a shows the average trajectories for each latent class for boys; Figure 1b is for girls. The identified classes were similar for each sex and were thus the same nomenclature was used.

- The largest class (47.7% of boys; 38.0% of girls) and second largest class (23.5% of boys; 30.7% of girls) had average trajectories that were consistently in the normal weight range. These classes are referred to as “lower normal weight” and “higher normal weight”, respectively.
- The next largest class (17.1% of boys; 19.5% of girls) had an average trajectory that started in the normal weight range but ended in the overweight range. This class is referred to as “normal weight increasing to overweight”.
- The next largest class (8.4% of boys; 6.3% of girls) had an average trajectory that started in the overweight range but ended in the obese range. This class is referred to as “overweight increasing to obesity”.
- The final and smallest class (3.3% of boys; 5.5% of girls) had a mean trajectory that started in the overweight range but ended in the normal weight range. This class is referred to as “overweight decreasing to normal weight”.

Supplementary Figures 3a to 4e show the average fitted trajectories and individual observed trajectories for each class. And Supplementary Figures 5a to 6e show the distributions of the posterior probabilities for assigned class membership.

Using model class assignments, more of the 2001 MCS children than the 1958 NCDS children were in the “normal weight increasing to overweight” class (e.g., 12.4 vs 3.5% for boys) and the “overweight increasing to obesity” class (e.g., 22.1 vs 13.5% for boys) (Table 2). Consequently, and as shown in Table 3, children in the 2001 MCS (compared to the 1958 NCDS) were estimated to have much higher odds of being in these classes (compared to the “lower normal weight” class). For example, girls in the 2001 MCS had 16.44 (11.87, 22.77) times higher odds of being the “overweight increasing to obesity” class and 4.57 (3.82, 4.45) times higher odds of being the “normal weight increasing to overweight” class. Children in the 2001 MCS also had higher odds of being in the “higher normal weight” and “overweight decreasing to normal weight” classes, although these estimates were considerably smaller.

### Parental exposures and class membership

The fully adjusted estimates of the relationships of maternal and paternal height (cm/10), BMI, and weight status with class membership are shown in Table 4. The unadjusted and adjusted for birth cohort estimates are shown in Supplementary Tables 10 & 11. There was limited evidence that maternal or paternal height was associated with class membership, with the exception that, in boys, a 10 cm increase in maternal height was related to 0.86 (0.74, 0.99) times lower odds of being in the “overweight increasing to obesity” class (Table 4). Conversely, maternal BMI and paternal BMI were positively related to class membership in all instances, with the estimates being strongest for the “overweight increasing to obesity” contrast followed by the “normal weight increasing to overweight” contrast. As shown in Supplementary Table 12, there was limited evidence that these associations for parental BMI differed between the two birth cohorts. There was, however, some evidence that maternal obesity was more strongly related to odds of being in the “overweight increasing to obesity” class than paternal obesity (Table 4). For example, in boys, maternal obesity was associated with 11.65 (8.39, 16.17) times higher odds while paternal obesity was associated with 6.08 (4.18, 8.84) times higher odds.

Table 5 examines maternal and paternal BMI together in fully adjusted models. All the estimates were positive, with 95% CIs that did not cross one, indicating that both maternal BMI and paternal BMI were associated with class membership. There was no evidence of an interaction between maternal and paternal BMI suggesting no departure from multiplicativity. However, all RERI estimates were negative, with confidence intervals that did not cross zero, thereby providing evidence of negative departure from additivity. When considering maternal and paternal weight statuses together, the odds of being in the “overweight increasing to obesity” class was 9.01 (6.37, 12.75) times higher for boys, and 13.11 (8.74, 19.66) times higher for girls, whose parents both had overweight or obesity compared to children whose parents both had normal weight (Table 6). Supplementary Table 13 shows the estimates using the following different groupings: both parents had normal weight, one parent had overweight, both parents had overweight, one or both parents had obesity.

### Socioeconomic position and class membership

As shown in Supplementary Table 14, lower socioeconomic position (for each of the three variables considered) was associated with higher odds of belonging to the “overweight increasing to obesity” class (compared to the largest normal weight class).

## Discussion

This paper demonstrates the application of growth mixture modelling to serial BMI data pooled from two birth cohort studies, one born in 1958 before the obesity epidemic and one born in 2001 during the obesity epidemic. By using this approach, we were able to describe the secular change in paediatric BMI trajectories in more realistic and holistic terms than just a change in the mean BMI or levels of overweight and obesity. Only a handful of other studies have employed this theoretically interesting and realistic analytical strategy.[7, 12, 38] Most strikingly, we found that more than 1 in 10 children in the 2001 MCS were in the most deleterious “overweight increasing to obesity” class, compared to less than 1 in 30 in the 1958 NCDS. The obesity epidemic in the UK is therefore explained, at least in part, by a dramatic increase in the number of pre-pubertal children with overweight becoming obese during adolescence. This is particularly worrying given that adolescent BMI gains are more strongly related (than childhood BMI gains) to increases in visceral adiposity,[39] tissue which plays a role in many pathological processes. Indeed, BMI increase during puberty is more strongly related to cardiovascular mortality than BMI during childhood.[40]

Maternal and paternal BMI were associated with latent class membership, with evidence of negative departure from additivity. This means that the combined effect of maternal and paternal BMI was smaller than the sum of the individual effects of these two exposures. Conversely, we found no evidence of departure from multiplicativity. This is in agreement with previous studies, including those using data from the 1970 British Cohort Study (BCS).[10, 20] This other nationally representative cohort was not used in the present analysis because BMI was only available at 10 and 16 years of age, nearly one-third of the 16-year measurements were self-reported, and the response rate at 16 years was low (for reasons that have been explained elsewhere).[20] Our reported estimates for maternal BMI and paternal BMI were generally of a similar magnitude, suggesting that, if causal, both exposures are equally important targets to stop the intergenerational transmission of high BMI. Similarly, investigating latent patterns of BMI development between 10-42 years of age in the 1970 BCS, Viner et al reported that maternal and paternal BMI were related to 1.10 (1.07, 1.13) and 1.08 (1.04, 1.13), respectively, times higher odds of their child belonging to an “adolescent and young adult-onset obesity” class compared to a “normative” class.[10] These findings do not support the foetal overnutrition hypothesis that, if the intrauterine environment is an independent factor for offspring obesity development (e.g., due to long-lasting biological effects of maternal adiposity during pregnancy on foetal energy metabolism and the endocrine system), the effect of maternal BMI will be stronger than that for paternal BMI.[41] We did find some evidence that maternal obesity was more strongly related to odds of being in the “overweight increasing to obesity” class than paternal obesity, particularly for boys. However, ORs are on a relative scale and this finding is influenced by a lower percentage of fathers than mothers having normal weight (due to a higher percentage of fathers than mothers having overweight).[42] While some individual studies have found maternal BMI to be more strongly related to childhood BMI than paternal BMI,[43, 44] a published systematic review of the literature found limited evidence to support this proposition and thus the foetal overnutrition hypothesis.[45]

The most comparable study to ours was conducted by Nedelec et al and published in this journal in 2021.[7] Those authors developed a sex-combined growth mixture model to describe latent patterns of BMI Z-score change between 2-18 years of age using data from 12,040 Finnish children born in either 1966 or 1986. Of the four identified latent classes, the most obesogenic comprised 3.7% of the sample and demonstrated an average trajectory that increased from approximately the 85^th^ (internal) centile to above the 99^th^ centile by 7 years of age, before plateauing. Conversely, for each sex, our “overweight increasing to obesity” class was near the 90^th^ centile of the IOTF charts at 7 years of age, increasing to approximately the 99^th^ centile by 17 years of age. The most recent cohort in our study was born in 2001, while the most recent cohort in the Nedelec et al study was born in 1986. Given what we know about secular trends in BMI trajectories,[11] it makes sense that our most obesogenic trajectory class (which mainly comprised children born in 2001) 1) crossed upwards though the centiles while Nedelec et al’s did not and 2) comprised 8.4% of boys and 6.3% of girls while Nedelec et al’s comprised only 3.7% of boys and girls. The other classes were also not similar between the two studies. In addition to the different populations being studied and the different growth charts used for comparison, this is likely to reflect quite different growth mixture modelling strategies.[46, 47] Further, Nedelec et al only investigated maternal BMI (and not weight status) while we were able to consider the individual and combined associations of maternal and paternal BMI (and weight status) with class membership.

While there are known biological mechanisms through which maternal BMI can influence offspring BMI (e.g., placental function and altered breast milk composition),[48–50] the associations observed in the present study between parental BMI and latent patterns of offspring BMI development are also due to the complex interplay of genetics, epigenetics, and the shared family environment. BMI between 7-17 years of age is highly heritable and there will not have been any change in the gene pool during the studied time period, perhaps except for that due to an increase in ethnic diversity.[51, 52] We do, however, know that genetic variants for obesity have stronger effects in obesogenic environments.[53, 54] Such gene-by-environment interactions may partly underlie the strong reported associations of parental obesity with membership of the, predominately 2001 cohort, “overweight increasing to obesity” class. We also know that assortative mating for BMI has increased alongside the obesity epidemic.[55] This phenomenon may have increased genetic predisposition to obesity in the 2001 cohort children, but the (theoretically) stronger tendency of a mother and father to have similar BMI values in the 2001 (compared to 1958) cohort could also influence offspring BMI due to non-genetic factors.[56] Our analysis investigating the associations of parental BMI with latent patterns of offspring BMI development did adjust for traditional confounders, but we did not consider the mediating role of the shared family environment. This is a particularly important consideration for future work as it is modifiable.

The main strength of the paper lies in the data (i.e., 25931 children, with serial objective BMI measurements, from two birth cohorts) and meticulous development of the growth mixture model. Our model development process considered several age functions for the trajectory shape, removal of default constraints on the growth term variances and covariances, different specifications of the within-class residual variance/error structure, and different autocorrelation structures.[46] Unlike the majority of other BMI growth mixture modelling papers in the literature,[57] our analysis addressed all of the points on the Guidelines for Reporting on Latent Trajectory Studies (GRoLTS) Checklist.[58] As the specification of growth mixture models become more refined, it is common for model fit to improve (e.g., lower BIC) but for the degree of separation between the classes to deteriorate (e.g., lower entropy).[47] This is exactly what we observed, and we acknowledge the limitation that our final models had low entropy (0.55 for boys; 0.52 for girls). We did, however, properly account for the uncertainty in class membership by using the 3-step BCH method of investigating auxiliary variables. Simulation studies have shown that, except in large sample sizes (N≥10,000), estimates (e.g., of associations of auxiliary variables with class membership) using the BCH approach can be biased in situations where the entropy is very low (≤0.5).[59, 60] The entropy in our models was low, but our estimates our unlikely to be biased (due to uncertainty in class assignment) because our sample size was very large (N~13,000 for each growth mixture model). A common mistake (according to van de Schoot et al) is to consider entropy during model development, ultimately leading to choosing a final model that has good entropy but poor model fit and less informative classes.[58] We did not make this mistake and are confident that our classes approximately reflect “real”, for want of another word, sub-populations of children.

Most of the paternal weight and height data were self-reported. Because people tend to overestimate their height and underestimate their weight, particularly if they are female and/or have obesity,[61] corresponding BMI is underestimated. As a result, our estimates might be biased away from the null; the true effects of maternal and paternal BMI on offspring latent BMI class membership might be weaker than reported in the present paper. While both the 1958 NCDS and 2001 MCS were designed to be nationally representative, our sample was limited to approximately 70% of each cohort. This is a relatively large proportion compared to many other published studies using these cohorts. Nonetheless, we do acknowledge that differential selection into our sample may have also biased results.[62] Finally, BMI is a far from perfect index and indicator of adiposity.[63, 64] Some of the differences between classes in mean BMI trajectories might reflect underlying differences in child height, pubertal timing, and body composition. It is also reasonable to question whether part of the observed associations of parental BMI with offspring latent BMI class membership might be driven by intergenerational transmission of stature. However, we think this is unlikely given that 1) height and BMI are negatively correlated in adulthood but positively correlated in much of childhood and adolescence [63] and 2) maternal and paternal height were not strongly associated with class membership in our analyses.

In conclusion, from before to well during the obesity epidemic era in the UK, there has been a four-fold increase in the proportion of children belonging to a sub-population characterised by overweight at 7 years progressing to obesity at 17 years. Our results implicate excess parental BMI as a correlate of this secular change because 1) more than one-third of the mothers and fathers of this sub-population had obesity (and more than one additional third had overweight but not obesity) and 2) maternal and paternal obesity were associated with very higher odds of their children belonging to this sub-population. There has also been a two-fold increase in the proportion of children belonging to a larger sub-population characterised by normal weight at 7 years progressing to overweight at 17 years, but this group is unlikely to represent the “low-hanging fruit” or priority for targeted intervention programmes. In addition to providing further evidence on the need to break the strong intergenerational transmission of obesity risk, our findings emphasise the need for a national adolescent weight gain monitoring programme.

## Supplementary Material

SUPPLEMENTAL MATERIAL

## Figures and Tables

**Table 1:** Description of the study sample

		Boys				Girls			
		Total (N=13220)		1958 NCDS (N=6727)	2001 MCS (N=6493)	Total (N=12711)		1958 NCDS (N=6336)	2001 MCS (N=6375)
			% missing				% missing		
Cohort			0				0		
1958 NCDS	N (%)	6727 (50.9)				6336 (49.9)			
2001 MCS	N (%)	6493 (49.1)				6375 (50.1)			
Ethnicity			1.2				0.7		
White British	N (%)	11849 (90.7)		6435 (98.0)	5414 (83.4)	11422 (90.5)		6129 (98.1)	5293 (83.0)
Other	N (%)	1209 (9.3)		130 (2.0)	1079 (16.6)	1200 (9.5)		118 (1.9)	1082 (17.0)
Mother									
Age (years)	Mean (SD)	28.2 (5.8)	5.7	27.5 (5.7)	28.9 (5.8)	28.2 (5.8)	5.0	27.5 (5.6)	28.9 (5.8)
Height (cm)	Mean (SD)	162.3 (6.8)	5.3	161.1 (6.4)	163.5 (6.9)	162.4 (6.8)	4.8	161.1 (6.4)	163.7 (7.0)
BMI (kg/m^2^)	Median (IQR)	24.0 (21.8, 27.3)	17.3	23.6 (21.5, 26.3)	24.8 (22.3, 28.5)	24.0 (21.8, 27.1)	16.8	23.4 (21.5, 26.0)	24.9 (22.3, 28.5)
Underweight	N (%)	285 (2.6)		203 (3.4)	82 (1.7)	293 (2.8)		204 (3.5)	89 (1.9)
Normal weight	N (%)	6195 (56.7)		3726 (61.4)	2469 (50.8)	6056 (57.3)		3652 (63.4)	2404 (49.9)
Overweight	N (%)	3022 (27.7)		1618 (26.7)	1404 (28.9)	2882 (27.3)		1439 (25.0)	1443 (30.0)
Obese	N (%)	1426 (13.1)		520 (8.6)	906 (18.6)	1346 (12.7)		465 (8.1)	881 (18.3)
Father									
Age (years)	Mean (SD)	31.3 (6.3)	17.8	30.5 (6.4)	32.2 (6.0)	31.4 (6.2)	17.8	30.6 (6.3)	32.4 (6.0)
Height (cm)	Mean (SD)	175.7 (7.6)	29.2	174.5 (7.4)	177.8 (7.3)	175.8 (7.6)	29.1	174.5 (7.5)	177.9 (7.4)
BMI (kg/m^2^)	Median (IQR)	25.1 (23.1, 27.6)	31.8	24.5 (22.6, 26.6)	26.8 (24.5, 29.7)	25.2 (23.1, 27.7)	31.7	24.5 (22.6, 26.6)	26.8 (24.5, 29.4)
Underweight	N (%)	65 (0.7)		54 (0.9)	11 (0.4)	67 (0.8)		51 (0.9)	16 (0.5)
Normal weight	N (%)	4299 (47.7)		3354 (56.8)	945 (30.4)	4107 (47.3)		3187 (57.2)	920 (29.5)
Overweight	N (%)	3625 (40.2)		2170 (36.8)	1455 (46.8)	3553 (40.9)		2032 (36.5)	1521 (48.8)
Obese	N (%)	1025 (11.4)		325 (5.5)	700 (22.5)	955 (11.0)		298 (5.4)	657 (21.1)
Tenure			6.8				5.8		
Own (outright or mortgage)	N (%)	5735 (46.6)		3511 (56.9)	2224 (36.2)	5601 (46.8)		3364 (57.2)	2237 (36.7)
Other	N (%)	6581 (53.4)		2655 (43.1)	3926 (63.8)	6370 (53.2)		2517 (42.8)	3853 (63.3)
Occupational class			16.0				16.1		
I (Professional)	N (%)	544 (4.9)		284 (4.7)	260 (5.2)	537 (5.0)		263 (4.6)	274 (5.6)
II (Managerial and technical)	N (%)	3326 (30.0)		1193 (19.6)	2133 (42.4)	3225 (30.1)		1153 (20.1)	2072 (42.0)
IIIN (Skilled non-manual)	N (%)	1329 (12.0)		639 (10.5)	690 (13.7)	1265 (11.9)		628 (11.0)	637 (12.9)
IIIM (Skilled manual)	N (%)	3702 (33.3)		2563 (42.2)	1139 (22.6)	3410 (32.0)		2318 (40.5)	1092 (22.2)
IV (Partly-skilled)	N (%)	1558 (14.0)		888 (14.6)	670 (13.3)	1582 (14.8)		879 (15.3)	703 (14.3)
V (Unskilled)	N (%)	645 (5.8)		506 (8.3)	139 (2.8)	640 (6.0)		488 (8.5)	152 (3.1)
Maternal age left full-time education			16.8				15.2		
≥ 23	N (%)	407 (3.7)		26 (0.5)	381 (6.2)	418 (3.9)		16 (0.3)	402 (6.6)
21-22	N (%)	787 (7.2)		63 (1.3)	724 (11.8)	827 (7.7)		97 (2.1)	730 (12.0)
19-20	N (%)	536 (4.9)		63 (1.3)	473 (7.7)	525 (4.9)		69 (1.5)	456 (7.5)
18	N (%)	1090 (9.9)		112 (2.3)	978 (15.9)	1120 (10.4)		113 (2.4)	1007 (16.6)
17	N (%)	1075 (9.8)		213 (4.4)	862 (14.1)	1017 (9.4)		217 (4.6)	800 (13.2)
16	N (%)	2769 (25.2)		590 (12.1)	2179 (35.5)	2706 (25.1)		556 (11.9)	2150 (35.3)
15	N (%)	1892 (17.2)		1462 (30.0)	430 (7.0)	1858 (17.2)		1421 (30.3)	437 (7.2)
14	N (%)	2312 (21.0)		2258 (46.4)	54 (0.9)	2177 (20.2)		2126 (45.3)	51 (0.8)
≤ 13	N (%)	137 (1.2)		83 (1.7)	54 (0.9)	129 (1.2)		78 (1.7)	51 (0.8)

**Table 2:** Modal class membership tabulated against birth cohort, with column percentages, for each child sex

	Boys		Girls	
	1958 NCDS	2001 MCS	1958 NCDS	2001 MCS
Lower normal weight	3861 (57.4)	2447 (37.7)	3047 (48.1)	1780 (27.9)
Higher normal weight	1605 (23.9)	1505 (23.2)	1926 (30.4)	1981 (31.1)
Normal weight increasing to overweight	831 (12.4)	1432 (22.1)	913 (14.4)	1570 (24.6)
Overweight decreasing to normal weight	197 (2.9)	235 (3.6)	309 (4.9)	386 (6.1)
Overweight increasing to obesity	233 (3.5)	874 (13.5)	141 (2.2)	658 (10.3)

**Table 3:** Odds ratios for class membership according to birth cohort

		Lower normal weight (referent)	Higher normal weight		Normal weight increasing to overweight		Overweight decreasing to normal weight		Overweight increasing to obesity	
		%	%	OR (95% CI)	%	OR (95% CI)	%	OR (95% CI)	%	OR (95% CI)
Boys										
	Cohort									
	1958 NCDS (referent)	65.7	51.8	--	34.0	--	50.1	--	17.6	--
	2001 MCS	34.3	48.2	1.78 (1.45, 2.20)	66.0	3.72 (3.15, 4.39)	49.9	1.91 (1.45, 2.51)	82.4	8.99 (7.14, 11.33)
Girls										
	Cohort									
	1958 NCDS (referent)	70.7	47.0	--	34.6	--	49.0	--	12.8	--
	2001 MCS	29.3	53.0	2.72 (2.22, 3.34)	65.4	4.57 (3.82, 5.45)	51.0	2.51 (1.97, 3.19)	87.2	16.44 (11.87, 22.77)

Proportions and odds ratios weighted by estimated class probabilities using the 3-step BCH approach in Mplus

**Table 4:** Adjusted odds ratios for class membership according to maternal and paternal height, BMI, and weight status

		Lower normal weight (referent)	Higher normal weight		Normal weight increasing to overweight		Overweight decreasing to normal weight		Overweight increasing to obesity	
		$\bar{X}$ or %	$\bar{X}$ or	OR (95% CI)	$\bar{X}$ or	OR (95% CI)	$\bar{X}$ or	OR (95% CI)	$\bar{X}$ or	OR (95% CI)
Boys										
	Mother									
Model 1	Height (cm/10)	16.21	16.21	0.87 (0.73, 1.02)	16.27	0.98 (0.86, 1.11)	16.30	1.18 (0.96, 1.46)	16.23	0.86 (0.74, 0.99)
Model 2	BMI (kg/m^2^)	23.4	25.3	1.13 (1.09, 1.17)	26.1	1.16 (1.14, 1.19)	25.2	1.12 (1.08, 1.16)	28.9	1.25 (1.22, 1.29)
Model 3	Weight Status									
	Normal weight (referent)	71.8	57.6	--	48.7	--	55.7	--	26.4	--
	Overweight	22.0	30.0	1.73 (1.31, 2.24)	32.9	2.16 (1.76, 2.65)	30.1	1.69 (1.20, 2.38)	35.2	3.93 (3.01, 5.14)
	Obese	6.2	12.4	2.47 (1.64, 3.77)	18.4	3.82 (2.82, 5.19)	14.2	2.62 (1.58, 4.33)	38.4	11.65 (8.39, 16.17)
	Father									
Model 4	Height (cm/10)	17.53	17.61	1.01 (0.86, 1.19)	17.59	0.94 (0.83, 1.07)	17.56	1.05 (0.83, 1.31)	17.66	1.05 (0.89, 1.24)
Model 5	BMI (kg/m^2^)	24.6	25.6	1.08 (1.04, 1.13)	27.2	1.18 (1.14, 1.22)	25.9	1.11 (1.06, 1.17)	28.5	1.22 (1.18, 1.27)
Model 6	Weight Status									
	Normal weight (referent)	60.7	46.1	--	30.3	--	42.4	--	26.2	--
	Overweight	33.1	45.4	1.64 (1.27, 2.11)	49.7	2.53 (2.02, 3.17)	43.7	1.78 (1.25, 2.52)	38.9	2.03 (1.48, 2.78)
	Obese	6.2	8.5	1.39 (0.82, 2.38)	20.0	4.15 (2.96, 5.81)	13.9	2.73 (1.57, 4.76)	34.9	6.08 (4.18, 8.84)
Girls										
	Mother									
Model 7	Height (cm/10)	16.16	16.29	1.10 (0.94, 1.28)	16.27	1.08 (0.95, 1.23)	16.18	0.88 (0.71, 1.09)	16.31	1.12 (0.95, 1.33)
Model 8	BMI (kg/m^2^)	23.0	24.7	1.13 (1.09, 1.17)	26.9	1.24 (1.20, 1.28)	25.5	1.18 (1.14, 1.22)	29.5	1.31 (1.27, 1.35)
Model 9	Weight Status									
	Normal weight (referent)	76.1	62.9	--	40.0	--	54.8	--	26.7	--
	Overweight	18.7	28.5	1.86 (1.44, 2.41)	37.1	3.54 (2.82, 4.45)	30.5	2.22 (1.64, 3.01)	32.7	4.27 (3.08, 5.91)
	Obese	5.2	8.6	1.89 (1.18, 3.03)	22.9	6.78 (4.84, 9.50)	14.7	3.69 (2.37, 5.76)	40.6	14.16 (9.49, 21.13)
	Father									
Model 10	Height (cm/10)	17.51	17.65	1.06 (0.91, 1.23)	17.57	0.94 (0.82, 1.08)	17.62	1.08 (0.90, 1.30)	17.58	0.90 (0.74, 1.09)
Model 11	BMI (kg/m^2^)	24.3	25.6	1.10 (1.06, 1.15)	27.1	1.22 (1.18, 1.26)	26.0	1.15 (1.11, 1.20)	29.6	1.31 (1.26, 1.37)
Model 12	Weight Status									
	Normal weight (referent)	63.3	47.2	--	31.9	--	38.1	--	18.1	--
	Overweight	31.3	44.4	1.65 (1.29, 2.11)	49.4	2.57 (2.04, 3.23)	51.4	2.46 (1.83, 3.31)	40.9	3.00 (1.97, 4.56)
	Obese	5.4	8.4	1.47 (0.90, 2.40)	18.7	4.29 (2.99, 6.15)	10.5	2.52 (1.45, 4.37)	41.0	10.52 (6.62, 16.73)

Means/proportions and odds ratios weighted by estimated class probabilities using the 3-step BCH approach in Mplus, using FIML to handle missing data Models adjusted for ethnicity, maternal or paternal age, tenure, occupational class, maternal age left full-time education, and birth cohort

**Table 5:** Adjusted odds ratios for class membership according to maternal and paternal BMI and their interaction

		Lower normal weight (referent)	Higher normal weight	Normal weight increasing to overweight	Overweight decreasing to normal weight	Overweight increasing to obesity
			OR (95% CI)	OR (95% CI)	OR (95% CI)	OR (95% CI)
Boys						
Model 1	Mothers BMI (kg/m^2^)	--	1.12 (1.07, 1.17)	1.15 (1.12, 1.18)	1.11 (1.07, 1.15)	1.24 (1.20, 1.28)
	Fathers BMI (kg/m^2^)	--	1.07 (1.02, 1.12)	1.16 (1.12, 1.20)	1.11 (1.05, 1.17)	1.20 (1.16, 1.25)
	Interaction	--	0.99 (0.98, 1.01)	1.00 (0.99, 1.00)	1.00 (0.98, 1.01)	0.99 (0.98, 1.00)
	RERI		-0.20 (-0.26, -0.13)	-0.32 (-0.36, -0.27)	-0.22 (-0.29, -0.15)	-0.45 (-0.51, -0.38)
Girls						
Model 2	Mothers BMI (kg/m^2^)	--	1.12 (1.08, 1.17)	1.22 (1.18, 1.26)	1.17 (1.13, 1.21)	1.28 (1.24, 1.33)
	Fathers BMI (kg/m^2^)	--	1.09 (1.05, 1.14)	1.19 (1.15, 1.24)	1.14 (1.09, 1.19)	1.28 (1.22, 1.34)
	Interaction	--	0.99 (0.98, 1.00)	1.00 (0.99, 1.01)	1.00 (0.99, 1.01)	1.00 (0.99, 1.01)
	RERI		-0.23 (-0.29, -0.16)	-0.40 (-0.46, -0.34)	-0.31 (-0.37, -0.25)	-0.56 (-0.64, -0.49)

RERI: relative excess risk due to interaction Odds ratios weighted by estimated class probabilities using the 3-step BCH approach in Mplus, using FIML to handle missing data Models adjusted for ethnicity, maternal and paternal age, tenure, occupational class, maternal age left full-time education, and birth cohort Mothers BMI and Fathers BMI were each grand mean centered before computing the interaction term

**Table 6:** Adjusted odds ratios for class membership according to different combinations of maternal and paternal weight status

		Lower normal weight (referent)	Higher normal weight		Normal weight increasing to overweight		Overweight decreasing to normal weight		Overweight increasing to obesity	
		%	%	OR (95% CI)	%	OR (95% CI)	%	OR (95% CI)	%	OR (95% CI)
Boys										
Model 1	Both parents’ normal weight (referent)	49.9	32.8	--	25.4	--	32.1	--	15.5	--
	Mother (but not father) overweight or obese	17.3	24.1	2.15 (1.60, 2.90)	24.4	2.60 (2.03, 3.32)	21.8	1.82 (1.20, 2.77)	41.4	6.47 (4.83, 8.67)
	Father (but not mother) overweight or obese	23.2	26.6	1.71 (1.29, 2.25)	26.7	2.32 (1.84, 2.92)	24.2	1.64 (1.11, 2.42)	19.6	3.08 (2.23, 4.25)
	Both parents overweight or obese	9.6	16.5	2.64 (1.84, 3.79)	23.5	5.12 (3.88, 6.72)	21.9	3.53 (2.31, 5.39)	23.5	9.01 (6.37, 12.75)
Girls										
Model 2	Both parents’ normal weight (referent)	54.4	37.6	--	21.7	--	28.7	--	17.0	--
	Mother (but not father) overweight or obese	14.4	21.4	2.20 (1.63, 2.97)	29.1	4.65 (3.54, 6.09)	24.1	3.12 (2.19, 4.43)	35.9	6.51 (4.58, 9.26)
	Father (but not mother) overweight or obese	23.0	26.4	1.69 (1.31, 2.18)	22.9	2.69 (2.08, 3.49)	28.8	2.36 (1.71, 3.25)	18.7	3.17 (2.16, 4.65)
	Both parents overweight or obese	8.2	14.6	2.69 (1.88, 3.87)	26.3	8.73 (6.40, 11.91)	18.4	4.24 (2.81, 6.39)	28.4	13.11 (8.74, 19.66)

Odds ratios weighted by estimated class probabilities using the 3-step BCH approach in Mplus, using FIML to handle missing data Models adjusted for ethnicity, maternal and paternal age, tenure, occupational class, maternal age left full-time education, and birth cohort

## Data Availability

Available from the first author upon request.

## Data Availability Statement

The datasets analysed during the current study are available in the UK Data Archive repository, https://www.data-archive.ac.uk/.
