# Supplementary material for: The associations of maternal and paternal obesity with latent patterns of offspring BMI development between 7-17 years of age: pooled analyses of cohorts born in 1958 and 2001 in the United Kingdom
Source: Int J Obes (Lond). Author manuscript; Available in PMC 2023 Jan 16. (PMC9834052; doi:10.1038/s41366-022-01237-6)

Supplementary Table 1. Description of the longitudinal BMI data for boys

| Visit | Age (years) | | | | | | N (%) | | | BMI (kg/m^2^) | | |
| --- | --- | --- | --- | --- | --- | --- | --- | --- | --- | --- | --- | --- |
|  | 50^th^  centile | 25^th^  centile | 75^th^  centile | Min | Max | Range | Total | 1958 NCDS | 2001 MCS | 50^th^  centile | 25^th^  centile | 75^th^  centile |
| 1 | 7.3 | 7.2 | 7.4 | 6.5 | 8.5 | 2.0 | 11974 | 6034 (50.4) | 5940 (49.6) | 15.9 | 15.1 | 17.0 |
| 2 | 11.3 | 11.2 | 11.4 | 10.2 | 12.8 | 2.7 | 12120 | 6014 (49.6) | 6106 (50.4) | 17.4 | 16.1 | 19.4 |
| 3 | 14.3 | 14.0 | 14.5 | 13.1 | 15.4 | 2.3 | 5374 | 14 (0.3) | 5360 (99.7) | 20.0 | 18.2 | 22.8 |
| 4 | 15.8 | 15.7 | 15.9 | 15.5 | 16.5 | 1.0 | 5207 | 5172 (99.3) | 35 (0.7) | 19.9 | 18.5 | 21.4 |
| 5 | 17.2 | 16.9 | 17.4 | 16.5 | 18.3 | 1.8 | 4509 | 58 (1.3) | 4451 (98.7) | 21.9 | 19.8 | 25.2 |

Supplementary Table 2. Description of the longitudinal BMI data for girls

| Visit | Age (years) | | | | | | N (%) | | | BMI (kg/m^2^) | | |
| --- | --- | --- | --- | --- | --- | --- | --- | --- | --- | --- | --- | --- |
|  | 50^th^  centile | 25^th^  centile | 75^th^  centile | Min | Max | Range | Total | 1958 NCDS | 2001 MCS | 50^th^  centile | 25^th^  centile | 75^th^  centile |
| 1 | 7.3 | 7.2 | 7.4 | 6.3 | 8.5 | 2.2 | 11647 | 5686 (48.8) | 5961 (51.2) | 15.9 | 14.9 | 17.1 |
| 2 | 11.3 | 11.2 | 11.4 | 10.2 | 12.7 | 2.5 | 11770 | 5725 (48.6) | 6045 (51.4) | 17.8 | 16.2 | 20.2 |
| 3 | 14.2 | 14.0 | 14.5 | 13.2 | 15.4 | 2.2 | 5256 | 13 (0.3) | 5243 (99.7) | 21.1 | 19.1 | 23.9 |
| 4 | 15.8 | 15.7 | 15.9 | 15.5 | 16.5 | 1.0 | 4888 | 4863 (99.5) | 25 (0.5) | 20.6 | 19.0 | 22.5 |
| 5 | 17.2 | 16.9 | 17.4 | 16.5 | 18.2 | 1.7 | 4583 | 64 (1.4) | 4519 (98.6) | 22.4 | 20.2 | 26.6 |

Supplementary Table 3. Number (%) of children with 2, 3, or 4 serial BMI measurements

|  | Boys | | | Girls | | |
| --- | --- | --- | --- | --- | --- | --- |
| Number of serial  measurements | Total | 1958 NCDS | 2001 MCS | Total | 1958 NCDS | 2001 MCS |
| 2 | 4077 (30.8) | 2889 (42.9) | 1188 (18.3) | 3758 (29.6) | 2657 (41.9) | 1101 (17.3) |
| 3 | 5542 (41.9) | 3838 (57.1) | 1704 (26.2) | 5184 (40.8) | 3679 (58.1) | 1505 (23.6) |
| 4 | 3601 (27.2) |  | 3601 (55.5) | 3769 (29.7) |  | 3769 (59.1) |

Supplementary Table 4. Comparison of the BIC between growth mixture models with different specifications for boys

|  | Model 1 | Model 2 | | Model 3 | | Model 4 | |
| --- | --- | --- | --- | --- | --- | --- | --- |
|  | Linear  + Tscores | Linear  + Tscores  + residual variances  allowed to differ  across classes | | Quadratic  + Tscores  + residual variances  allowed to differ  across classes | | Quadratic  + Tscores  + residual variances  allowed to differ  across classes  + intercept variance  allowed to differ  across classes | |
|  |  |  | Δ from  model 1 |  | Δ from  model 2 |  | Δ from  model 3 |
| Class |  |  |  |  |  |  |  |
| 1 | 169614 | 169614 | 0 | 168136 | -1478 | 168136 | 0 |
| 2 | 166174 | 161387 | -4787 | 158888 | -2499 | 157937 | -951 |
| 3 | 164634 | 160147 | -4487 | 157156 | -2991 | 156773 | -383 |
| 4 | 163970 | 159761 | -4209 | 156671 | -3090 | 156385 | -286 |
| 5 | 163570 | 159576 | -3994 | 156395 | -3181 | 156228 | -167 |
| 6 | 163267 | 159491 | -3776 | 156313 | -3178 | 156128 | -185 |

Supplementary Table 5. Comparison of the BIC between growth mixture models with different specifications for girls

|  | Model 1 | Model 2 | | Model 3 | | Model 4 | |
| --- | --- | --- | --- | --- | --- | --- | --- |
|  | Linear  + Tscores | Linear  + Tscores  + residual variances  allowed to differ  across classes | | Quadratic  + Tscores  + residual variances  allowed to differ  across classes | | Quadratic  + Tscores  + residual variances  allowed to differ  across classes  + intercept variance  allowed to differ  across classes | |
|  |  |  | Δ from  model 1 |  | Δ from  model 2 |  | Δ from  model 3 |
| Class |  |  |  |  |  |  |  |
| 1 | 170468 | 170468 | 0 | 169794 | -674 | 169794 | 0 |
| 2 | 167673 | 164493 | -3180 | 163461 | -1032 | 162672 | -789 |
| 3 | 166471 | 163376 | -3095 | 162009 | -1367 | 161610 | -399 |
| 4 | 165822 | 163059 | -2763 | 161614 | -1445 | 161274 | -340 |
| 5 | 165523 | 162883 | -2640 | 161336 | -1547 | 161023 | -313 |
| 6 | 165250 | 162850 | -2400 | 161194 | -1656 | 160973 | -221 |

Supplementary Table 6. Summary of final mixture models for boys

| Classes | 1 | 2 | 3 | 4 | 5 | 6 |
| --- | --- | --- | --- | --- | --- | --- |
| AIC | 168031 | 157757 | 156518 | 156055 | 155824 | 155648 |
| BIC | 168136 | 157937 | 156773 | 156385 | 156228 | 156128 |
| BIC difference | -- | -10199 | -1164 | -388 | -157 | -100 |
| Entropy  . | -- | 0.762 | 0.637 | 0.607 | 0.549 | 0.583 |
| Posterior probability [mean] |  |  |  |  |  |  |
| Class-1 | -- | 0.944 | 0.882 | 0.670 | 0.727 | 0.663 |
| Class-2 | -- | 0.920 | 0.771 | 0.835 | 0.679 | 0.744 |
| Class-3 | -- |  | 0.857 | 0.679 | 0.613 | 0.681 |
| Class-4 | -- |  |  | 0.871 | 0.835 | 0.645 |
| Class-5 | -- |  |  |  | 0.730 | 0.726 |
| Class-6 | -- |  |  |  |  | 0.764 |
| Posterior probability > 0.7 [%] |  |  |  |  |  |  |
| Class-1 | -- | 95.5 | 82.1 | 40.5 | 61.2 | 41.9 |
| Class-2 | -- | 89.3 | 69.4 | 82.2 | 45.0 | 65.5 |
| Class-3 | -- |  | 86.0 | 44.4 | 25.3 | 44.0 |
| Class-4 | -- |  |  | 81.0 | 75.0 | 33.7 |
| Class-5 | -- |  |  |  | 56.0 | 54.5 |
| Class-6 | -- |  |  |  |  | 59.5 |
| Class membership [N (%)] |  |  |  |  |  |  |
| Class-1 | 13220 (100.0) | 10223 (77.3) | 1520 (11.5) | 986 (7.5) | 6308 (47.7) | 1533 (11.6) |
| Class-2 |  | 2997 (22.7) | 3689 (27.9) | 8125 (61.5) | 2263 (17.1) | 6575 (49.7) |
| Class-3 |  |  | 8011 (60.6) | 2698 (20.4) | 3110 (23.5) | 350 (2.6) |
| Class-4 |  |  |  | 1411 (10.7) | 1107 (8.4) | 3634 (27.5) |
| Class-5 |  |  |  |  | 432 (3.3) | 642 (4.9) |
| Class-6 |  |  |  |  |  | 486 (3.7) |

Supplementary Table 7. Summary of final mixture models for girls

| Classes | 1 | 2 | 3 | 4 | 5 | 6 |
| --- | --- | --- | --- | --- | --- | --- |
| AIC | 169689 | 162493 | 161357 | 160946 | 160621 | 160497 |
| BIC | 169794 | 162672 | 161610 | 161274 | 161023 | 160973 |
| BIC difference | -- | -7122 | -1062 | -336 | -251 | -50 |
| Entropy | -- | 0.709 | 0.608 | 0.577 | 0.524 | 0.480 |
| Posterior probability [mean] |  |  |  |  |  |  |
| Class-1 | -- | 0.930 | 0.874 | 0.840 | 0.674 | 0.543 |
| Class-2 | -- | 0.897 | 0.778 | 0.672 | 0.640 | 0.598 |
| Class-3 | -- |  | 0.137 | 0.820 | 0.699 | 0.598 |
| Class-4 | -- |  |  | 0.682 | 0.820 | 0.700 |
| Class-5 | -- |  |  |  | 0.703 | 0.659 |
| Class-6 | -- |  |  |  |  | 0.810 |
| Posterior probability > 0.7 [%] |  |  |  |  |  |  |
| Class-1 | -- | 93.8 | 81.2 | 75.3 | 45.0 | 15.4 |
| Class-2 | -- | 85.7 | 70.5 | 44.2 | 32.2 | 26.1 |
| Class-3 | -- |  | 82.1 | 79.4 | 49.2 | 24.7 |
| Class-4 | -- |  |  | 43.2 | 72.2 | 48.1 |
| Class-5 | -- |  |  |  | 54.6 | 42.1 |
| Class-6 | -- |  |  |  |  | 69.4 |
| Class membership [N (%)] |  |  |  |  |  |  |
| Class-1 | 12711 (100.0) | 9841 (77.4) | 1233 (9.7) | 1138 (9.0) | 2483 (19.5) | 1146 (9.0) |
| Class-2 |  | 2870 (22.6) | 4500 (35.4) | 3211 (25.3) | 3907 (30.7) | 3797 (29.9) |
| Class-3 |  |  | 6978 (54.9) | 7302 (57.4) | 695 (5.5) | 4042 (31.8) |
| Class-4 |  |  |  | 1060 (8.3) | 799 (6.3) | 617 (4.9) |
| Class-5 |  |  |  |  | 4827 (38.0) | 2340 (18.4) |
| Class-6 |  |  |  |  |  | 769 (6.1) |

Supplementary Table 8. Illustration of the combined parental weight status exposures for boys

|  |  |  | Father weight status | | | |
| --- | --- | --- | --- | --- | --- | --- |
|  |  |  | Missing | Normal weight | Overweight | Obese |
|  | Mother  weight  status | Missing | 1681 | 195 | 254 | 162 |
|  |  | Normal weight | 1310 | 2757 | 2007 | 406 |
|  |  | Overweight | 716 | 1036 | 995 | 275 |
|  |  | Obese | 499 | 376 | 369 | 182 |
| Exposure 2 |  | | | | | |
| Both parents’ normal weight | 4262 = 2757 + 1310 + 195 | | | | | |
| Mother (but not father) overweight or obese | 2627 = 376 + 499 + 1036 + 716 | | | | | |
| Father (but not mother) overweight or obese | 2829 = 406 + 162 + 2007 + 254 | | | | | |
| Both parents overweight or obese | 1821 = 182 + 369 + 275 + 995 | | | | | |
| Missing | 1681 | | | | | |
| Exposure 1 |  | | | | | |
| Both parents’ normal weight | 4262 = 2757 + 1310 + 195 | | | | | |
| One parent overweight | 4013 = 1036 + 716 + 2007 + 254 | | | | | |
| Both parents’ overweight | 995 | | | | | |
| One or both parents obese | 2269 = 369 + 376 + 499 + 275 + 406 + 162 + 182 | | | | | |
| Missing | 1681 | | | | | |

Supplementary Table 9. Illustration of the combined parental weight status exposures for girls

|  |  |  | Father weight status | | | |
| --- | --- | --- | --- | --- | --- | --- |
|  |  |  | Missing | Normal weight | Overweight | Obese |
|  | Mother  weight  status | Missing | 1522 | 200 | 291 | 121 |
|  |  | Normal weight | 1325 | 2720 | 1947 | 357 |
|  |  | Overweight | 726 | 952 | 930 | 274 |
|  |  | Obese | 456 | 302 | 385 | 203 |
| Exposure 1 |  | | | | | |
| Both parents’ normal weight | 4245 = 2720 + 1325 + 200 | | | | | |
| Mother (but not father) overweight or obese | 2436 = 302 + 456 + 952 + 726 | | | | | |
| Father (but not mother) overweight or obese | 2716 = 357 + 121 + 1947 + 291 | | | | | |
| Both parents overweight or obese | 1792 = 203 + 385 + 274 + 930 | | | | | |
| Missing | 1522 | | | | | |
| Exposure 2 |  | | | | | |
| Both parents’ normal weight | 4245 = 2720 + 1325 + 200 | | | | | |
| One parent overweight | 3916 = 952 + 726 + 1947 + 291 | | | | | |
| Both parents’ overweight | 930 | | | | | |
| One or both parents obese | 2098 = 385 + 302 + 456 + 274 + 357 + 121 + 203 | | | | | |
| Missing | 1522 | | | | | |

Supplementary Table 10. Odds ratios for class membership according to maternal and paternal height, BMI, and weight status: Unadjusted

|  |  | Lower  normal weight  (referent) | Higher  normal weight | Normal weight  increasing to  overweight | Overweight  decreasing to  normal weight | Overweight  increasing to  obesity |
| --- | --- | --- | --- | --- | --- | --- |
|  |  |  | OR (95% CI) | OR (95% CI) | OR (95% CI) | OR (95% CI) |
| Boys |  |  |  |  |  |  |
|  | Mother |  |  |  |  |  |
| Model 1 | Height (cm/10) | -- | 1.01 (0.93, 1.23) | 1.14 (1.01, 1.29) | 1.19 (0.96, 1.47) | 1.07 (0.93, 1.23) |
| Model 2 | BMI (kg/m^2^) | -- | 1.15 (1.11, 1.19) | 1.19 (1.16, 1.22) | 1.14 (1.09, 1.18) | 1.30 (1.26, 1.34) |
| Model 3 | Weight Status |  |  |  |  |  |
|  | Normal weight (referent) | -- | -- | -- | -- | -- |
|  | Overweight | -- | 1.76 (1.35, 2.28) | 2.19 (1.77, 2.63) | 1.80 (1.28, 2.53) | 4.42 (3.42, 5.71) |
|  | Obese | -- | 3.08 (1.86, 5.09) | 4.85 (3.45, 6.83) | 3.35 (1.97, 5.69) | 18.65 (12.94, 26.87) |
|  | Father |  |  |  |  |  |
| Model 4 | Height (cm/10) | -- | 1.15 (0.99, 1.33) | 1.11 (0.98, 1.25) | 1.04 (0.84, 1.29) | 1.24 (1.06, 1.46) |
| Model 5 | BMI (kg/m^2^) | -- | 1.10 (1.05, 1.15) | 1.24 (1.20, 1.28) | 1.13 (1.07, 1.20) | 1.31 (1.27, 1.36) |
| Model 6 | Weight Status |  |  |  |  |  |
|  | Normal weight (referent) | -- | -- | -- | -- | -- |
|  | Overweight | -- | 1.76 (1.36, 2.27) | 2.92 (2.34, 3.63) | 1.85 (1.30, 2.64) | 2.70 (2.00, 3.64) |
|  | Obese | -- | 1.80 (0.80, 4.02) | 6.89 (4.70, 10.10) | 3.88 (2.20, 6.85) | 12.15 (7.93, 18.61) |
| Girls |  |  |  |  |  |  |
|  | Mother |  |  |  |  |  |
| Model 7 | Height (cm/10) | -- | 1.28 (1.11, 1.47) | 1.26 (1.11, 1.42) | 1.06 (0.86, 1.31) | 1.34 (1.13, 1.58) |
| Model 8 | BMI (kg/m^2^) | -- | 1.15 (1.11, 1.19) | 1.28 (1.24, 1.32) | 1.19 (1.15, 1.23) | 1.37 (1.33, 1.42) |
| Model 9 | Weight Status |  |  |  |  |  |
|  | Normal weight (referent) | -- | -- | -- | -- | -- |
|  | Overweight | -- | 1.94 (1.50, 2.52) | 3.79 (3.04, 4.71) | 2.28 (1.68, 3.08) | 5.14 (3.77, 7.01) |
|  | Obese | -- | 2.20 (1.26, 3.82) | 8.92 (6.14, 12.95) | 4.01 (2.47, 6.52) | 23.29 (14.45, 35.11) |
|  | Father |  |  |  |  |  |
| Model 10 | Height (cm/10) | -- | 1.24 (1.08, 1.43) | 1.13 (1.00, 1.28) | 1.22 (1.01, 1.47) | 1.08 (0.91, 1.29) |
| Model 11 | BMI (kg/m^2^) | -- | 1.15 (1.10, 1.20) | 1.29 (1.25, 1.34) | 1.18 (1.12, 1.23) | 1.43 (1.38, 1.50) |
| Model 12 | Weight Status |  |  |  |  |  |
|  | Normal weight (referent) | -- | -- | -- | -- | -- |
|  | Overweight | -- | 1.91 (1.49, 2.44) | 3.01 (2.40, 3.76) | 2.65 (1.97, 3.57) | 4.63 (3.06, 7.00) |
|  | Obese | -- | 3.19 (1.49, 6.82) | 9.31 (5.42, 15.98) | 3.82 (1.79. 8.12) | 30.19 (16.49, 55.26) |

Odds ratios weighted by estimated class probabilities using the 3-step BCH approach in Mplus, using FIML to handle missing data

Supplementary Table 11. Odds ratios for class membership according to maternal and paternal height, BMI, and weight status: Adjusted for birth cohort

|  |  | Lower  normal weight  (referent) | Higher  normal weight | Normal weight  increasing to  overweight | Overweight  decreasing to  normal weight | Overweight  increasing to  obesity |
| --- | --- | --- | --- | --- | --- | --- |
|  |  |  | OR (95% CI) | OR (95% CI) | OR (95% CI) | OR (95% CI) |
| Boys |  |  |  |  |  |  |
|  | Mother |  |  |  |  |  |
| Model 1 | Height (cm/10) | -- | 0.95 (0.81, 1.10) | 0.97 (0.86, 1.10) | 1.13 (0.91, 1.39) | 0.80 (0.70, 0.93) |
| Model 2 | BMI (kg/m^2^) | -- | 1.13 (1.09, 1.18) | 1.16 (1.14, 1.19) | 1.12 (1.08, 1.16) | 1.26 (1.23, 1.30) |
| Model 3 | Weight Status |  |  |  |  |  |
|  | Normal weight (referent) | -- | -- | -- | -- | -- |
|  | Overweight | -- | 1.71 (1.32, 2.21) | 2.14 (1.75, 2.63) | 1.74 (1.23, 2.44) | 4.12 (3.16, 5.36) |
|  | Obese | -- | 2.50 (1.63, 3.84) | 3.84 (2.82, 5.22) | 2.79 (1.68, 4.63) | 13.05 (9.39, 18.13) |
|  | Father |  |  |  |  |  |
| Model 4 | Height (cm/10) | -- | 1.06 (0.91, 1.24) | 0.94 (0.83, 1.06) | 0.98 (0.78, 1.21) | 0.88 (0.75, 1.04) |
| Model 5 | BMI (kg/m^2^) | -- | 1.09 (1.04, 1.13) | 1.17 (1.14, 1.21) | 1.11 (1.06, 1.17) | 1.21 (1.17, 1.25) |
| Model 6 | Weight Status |  |  |  |  |  |
|  | Normal weight (referent) | -- | -- | -- | -- | -- |
|  | Overweight | -- | 1.67 (1.30, 2.15) | 2.55 (2.04, 3.20) | 1.77 (1.25, 2.51) | 1.70 (1.25, 2.32) |
|  | Obese | -- | 1.48 (0.86, 2.56) | 4.19 (2.99, 5.89) | 2.85 (1.66, 4.89) | 5.05 (3.49, 7.32) |
| Girls |  |  |  |  |  |  |
|  | Mother |  |  |  |  |  |
| Model 7 | Height (cm/10) | -- | 1.12 (0.97, 1.30) | 1.03 (0.91, 1.17) | 0.94 (0.77, 1.15) | 0.97 (0.82, 1.15) |
| Model 8 | BMI (kg/m^2^) | -- | 1.13 (1.09, 1.17) | 1.24 (1.21, 1.28) | 1.17 (1.13, 1.21) | 1.33 (1.28, 1.37) |
| Model 9 | Weight Status |  |  |  |  |  |
|  | Normal weight (referent) | -- | -- | -- | -- | -- |
|  | Overweight | -- | 1.86 (1.44, 2.41) | 3.58 (2.86, 4.49) | 2.14 (1.59, 2.89) | 4.63 (3.35, 6.39) |
|  | Obese | -- | 1.88 (1.16, 3.05) | 7.04 (5.00, 9.90) | 3.46 (2.21, 5.42) | 16.57 (11.10, 24.73) |
|  | Father |  |  |  |  |  |
| Model 10 | Height (cm/10) | -- | 1.10 (0.95, 1.27) | 0.91 (0.80, 1.04) | 1.08 (0.91, 1.30) | 0.73 (0.61, 0.87) |
| Model 11 | BMI (kg/m^2^) | -- | 1.11 (1.06, 1.15) | 1.21 (1.17, 1.25) | 1.14 (1.09, 1.18) | 1.30 (1.25, 1.35) |
| Model 12 | Weight Status |  |  |  |  |  |
|  | Normal weight (referent) | -- | -- | -- | -- | -- |
|  | Overweight | -- | 1.68 (1.32, 2.14) | 2.52 (2.01, 3.17) | 2.39 (1.78, 3.21) | 2.54 (1.70, 3.79) |
|  | Obese | -- | 1.64 (1.00, 2.68) | 4.33 (3.02, 6.23) | 2.32 (1.28, 4.18) | 9.21 (5.92, 14.32) |

Odds ratios weighted by estimated class probabilities using the 3-step BCH approach in Mplus, using FIML to handle missing data

Supplementary Table 12. Odds ratios for class membership according to maternal and paternal BMI, testing for effect modification by birth cohort: Unadjusted

|  |  | Lower  normal weight  (referent) | Higher  normal weight | Normal weight  increasing to  overweight | Overweight  decreasing to  normal weight | Overweight  increasing to  obesity |
| --- | --- | --- | --- | --- | --- | --- |
|  |  |  | OR (95% CI) | OR (95% CI) | OR (95% CI) | OR (95% CI) |
| Boys |  |  |  |  |  |  |
|  | Mothers |  |  |  |  |  |
| Model 1 | BMI (kg/m^2^) effects in 1958 NCDS cohort | -- | 1.14 (1.09, 1.18) | 1.17 (1.14, 1.21) | 1.12 (1.07, 1.16) | 1.32 (1.28, 1.36) |
|  | BMI (kg/m^2^) effects in 2001 MCS cohort | -- | 1.13 (1.09, 1.17) | 1.15 (1.12, 1.18) | 1.13 (1.08, 1.17) | 1.25 (1.21, 1.29) |
|  | Difference (i.e., BMI x Cohort interaction) | -- | 0.99 (0.97, 1.01) | 0.99 (0.97, 1.00) | 1.01 (0.98, 1.04) | 0.95 (0.93, 0.96) |
|  | Fathers |  |  |  |  |  |
| Model 2 | BMI (kg/m^2^) effects in 1958 NCDS cohort | -- | 1.09 (1.04, 1.14) | 1.19 (1.15, 1.23) | 1.12 (1.06, 1.18) | 1.29 (1.24, 1.34) |
|  | BMI (kg/m^2^) effects in 2001 MCS cohort | -- | 1.08 (1.04, 1.13) | 1.17 (1.13, 1.20) | 1.10 (1.05, 1.16) | 1.22 (1.18, 1.27) |
|  | Difference (i.e., BMI x Cohort interaction) | -- | 1.00 (0.98, 1.01) | 0.98 (0.97, 1.00) | 0.99 (0.96, 1.01) | 0.95 (0.94, 0.97) |
| Girls |  |  |  |  |  |  |
|  | Mothers |  |  |  |  |  |
| Model 3 | BMI (kg/m^2^) effects in 1958 NCDS cohort | -- | 1.13 (1.08, 1.18) | 1.26 (1.22, 1.31) | 1.18 (1.14, 1.23) | 1.39 (1.34, 1.45) |
|  | BMI (kg/m^2^) effects in 2001 MCS cohort | -- | 1.12 (1.08, 1.16) | 1.22 (1.18, 1.26) | 1.16 (1.12, 1.20) | 1.31 (1.26, 1.35) |
|  | Difference (i.e., BMI x Cohort interaction) | -- | 0.99 (0.97, 1.01) | 0.97 (0.95, 0.99) | 0.98 (0.95, 1.01) | 0.94 (0.92, 0.95) |
|  | Fathers |  |  |  |  |  |
| Model 4 | BMI (kg/m^2^) effects in 1958 NCDS cohort | -- | 1.11 (1.06, 1.16) | 1.23 (1.19, 1.28) | 1.15 (1.10, 1.20) | 1.40 (1.34, 1.48) |
|  | BMI (kg/m^2^) effects in 2001 MCS cohort | -- | 1.10 (1.06, 1.15) | 1.20 (1.16, 1.24) | 1.13 (1.08, 1.18) | 1.33 (1.27, 1.38) |
|  | Difference (i.e., BMI x Cohort interaction) | -- | 0.99 (0.98, 1.01) | 0.98 (0.96, 0.99) | 0.98 (0.96, 1.01) | 0.94 (0.93, 0.96) |

Odds ratios weighted by estimated class probabilities using the 3-step BCH approach in Mplus, using FIML to handle missing data

Supplementary Table 13. Adjusted odds ratios for class membership according to different combinations of maternal and paternal weight status

|  |  | Lower  normal weight  (referent) | Higher  normal weight | |  | Normal weight  increasing to  overweight |  | Overweight  decreasing to  normal weight |  | Overweight  increasing to  obesity |
| --- | --- | --- | --- | --- | --- | --- | --- | --- | --- | --- |
|  |  | % | % | OR (95% CI) | % | OR (95% CI) | % | OR (95% CI) | % | OR (95% CI) |
| Boys |  |  |  |  |  |  |  |  |  |  |
| Model 1 | Both parents normal  weight (referent) | 50.0 | 32.7 | -- | 25.2 | -- | 31.9 | -- | 15.8 | -- |
|  | One parent overweight | 33.2 | 39.6 | 1.85 (1.44, 2.38) | 34.4 | 2.12 (1.71, 2.63) | 32.9 | 1.56 (1.09, 2.22) | 28.6 | 2.96 (2.20, 3.96) |
|  | Both parents overweight | 6.1 | 10.1 | 2.66 (1.73, 4.09) | 11.3 | 4.21 (3.01, 5.89) | 12.1 | 3.21 (1.91, 5.39) | 7.7 | 5.49 (3.41, 8.84) |
|  | One or both parents obese | 10.7 | 17.6 | 2.35 (1.64, 3.36) | 29.1 | 4.66 (3.59, 6.04) | 23.1 | 3.09 (2.03, 4.69) | 47.9 | 11.11 (8.19, 15.09) |
| Girls |  |  |  |  |  |  |  |  |  |  |
| Model 2 | Both parents normal  weight (referent) | 54.5 | 37.6 | -- | 21.6 | -- | 28.5 | -- | 17.2 | -- |
|  | One parent overweight | 30.7 | 39.5 | 1.95 (1.54, 2.46) | 35.0 | 3.02 (2.38, 3.83) | 42.5 | 2.67 (1.99, 3.57) | 25.5 | 2.89 (2.03, 4.10) |
|  | Both parents overweight | 5.7 | 8.8 | 2.49 (1.62, 3.83) | 12.4 | 6.52 (4.53, 9.38) | 8.6 | 3.01 (1.80, 5.05) | 6.6 | 5.19 (2.95, 9.12) |
|  | One or both parents obese | 9.1 | 14.1 | 2.10 (1.47, 3.00) | 31.0 | 7.41 (5.54, 9.92) | 20.4 | 3.91 (2.63, 5.83) | 50.7 | 13.77 (9.60, 19.73) |

Odds ratios weighted by estimated class probabilities using the 3-step BCH approach in Mplus, using FIML to handle missing data

Models adjusted for ethnicity, maternal and paternal age, tenure, occupational class, maternal age left full-time education, and birth cohort

Supplementary Table 14. Odds ratios for class membership according to socioeconomic position variables: Adjusted for birth cohort

|  |  | Lower  normal weight  (referent) | Higher  normal weight | Normal weight  increasing to  overweight | Overweight  decreasing to  normal weight | Overweight  increasing to  obesity |
| --- | --- | --- | --- | --- | --- | --- |
|  |  |  | OR (95% CI) | OR (95% CI) | OR (95% CI) | OR (95% CI) |
| Boys |  |  |  |  |  |  |
| Model 1 | Tenure |  |  |  |  |  |
|  | Own (outright or mortgage) (referent) | -- | -- | -- | -- | -- |
|  | Other | -- | 0.86 (0.69, 1.06) | 0.99 (0.83, 1.17) | 0.96 (0.72, 1.30) | 1.65 (1.36, 2.00) |
| Model 2 | Occupational class: ridit score | -- | 0.70 (0.46, 1.07) | 0.85 (0.62, 1.16) | 1.33 (0.78, 2.27) | 2.39 (1.66, 3.45) |
| Model 3 | Maternal age left full-time education: ridit score | -- | 0.63 (0.36, 1.11) | 1.51 (1.00, 2.27) | 1.81 (0.84, 3.91) | 3.37 (2.14, 5.29) |
| Girls |  |  |  |  |  |  |
| Model 1 | Tenure |  |  |  |  |  |
|  | Own (outright or mortgage) (referent) | -- | -- | -- | -- | -- |
|  | Other | -- | 0.81 (0.66, 0.99) | 1.24 (1.04, 1.48) | 0.73 (0.57, 0.94) | 1.96 (1.57, 2.45) |
| Model 2 | Occupational class: ridit score | -- | 0.69 (0.46, 1.03) | 1.63 (1.17, 2.28) | 0.80 (0.49, 1.31) | 3.42 (2.21, 5.29) |
| Model 3 | Maternal age left full-time education: ridit score | -- | 0.72 (0.42, 1.21) | 2.08 (1.34, 3.22) | 0.84 (0.47, 1.52) | 4.79 (2.83, 8.11) |

Odds ratios weighted by estimated class probabilities using the 3-step BCH approach in Mplus, using FIML to handle missing data

Supplementary Figure 1a. Final mixture model trajectories for a 1-class solution for boys


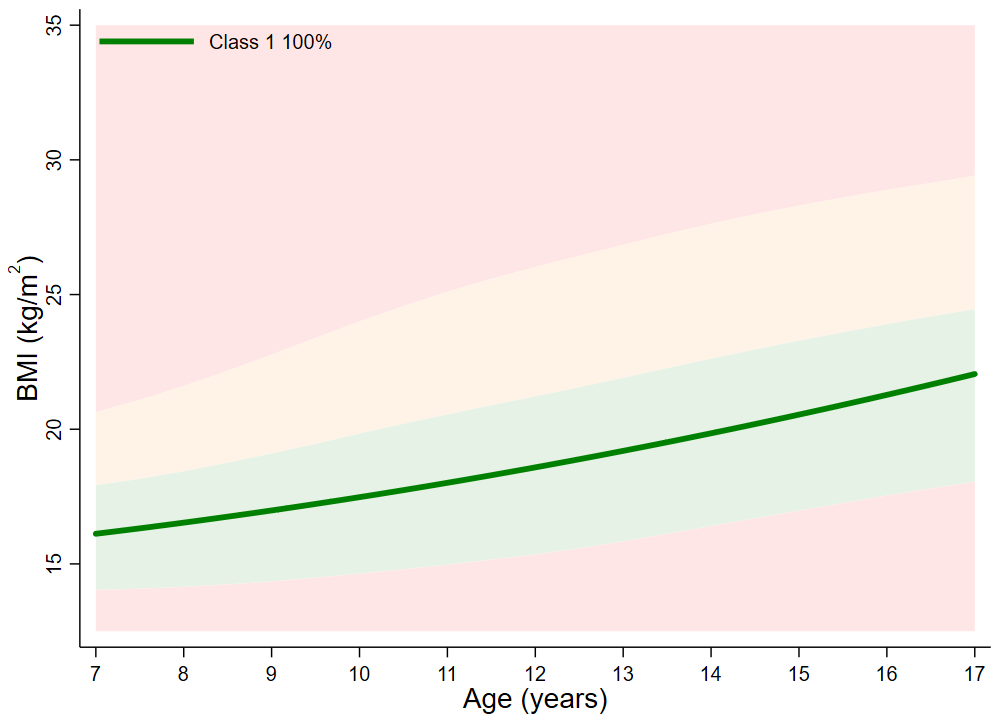


Supplementary Figure 1b. Final mixture model trajectories for a 2-class solution for boys


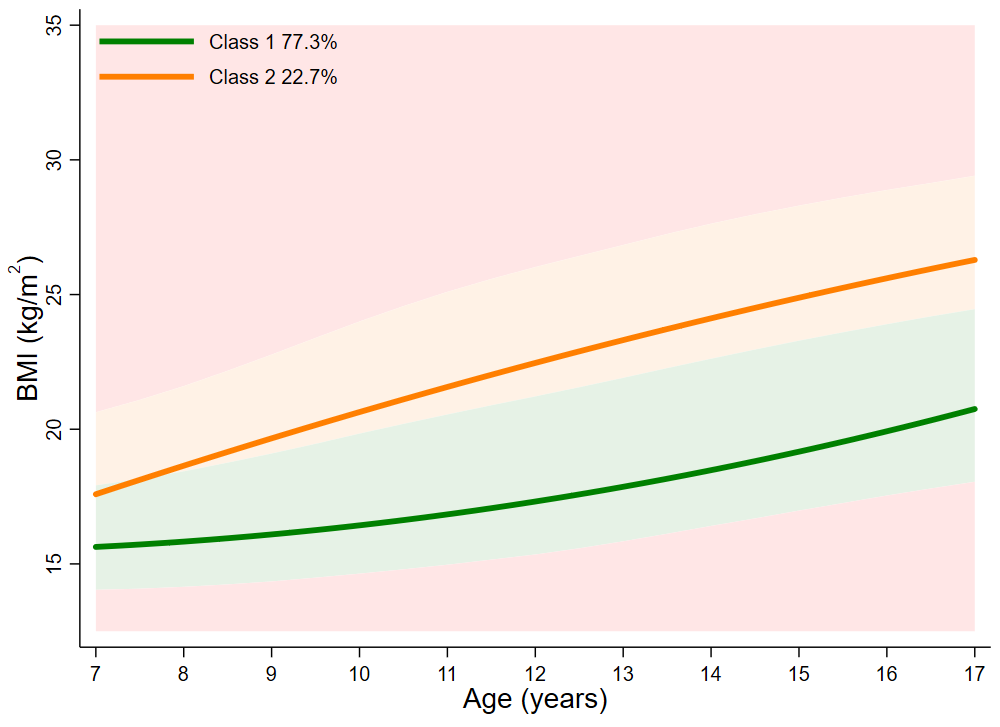


Supplementary Figure 1c. Final mixture model trajectories for a 3-class solution for boys


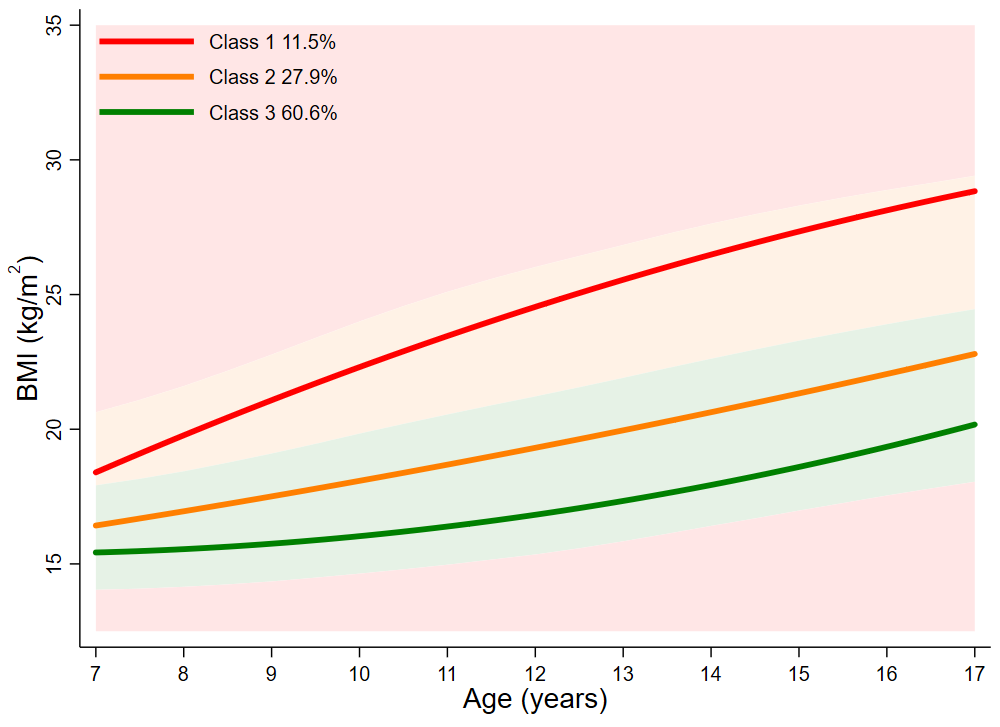


Supplementary Figure 1d. Final mixture model trajectories for a 4-class solution for boys


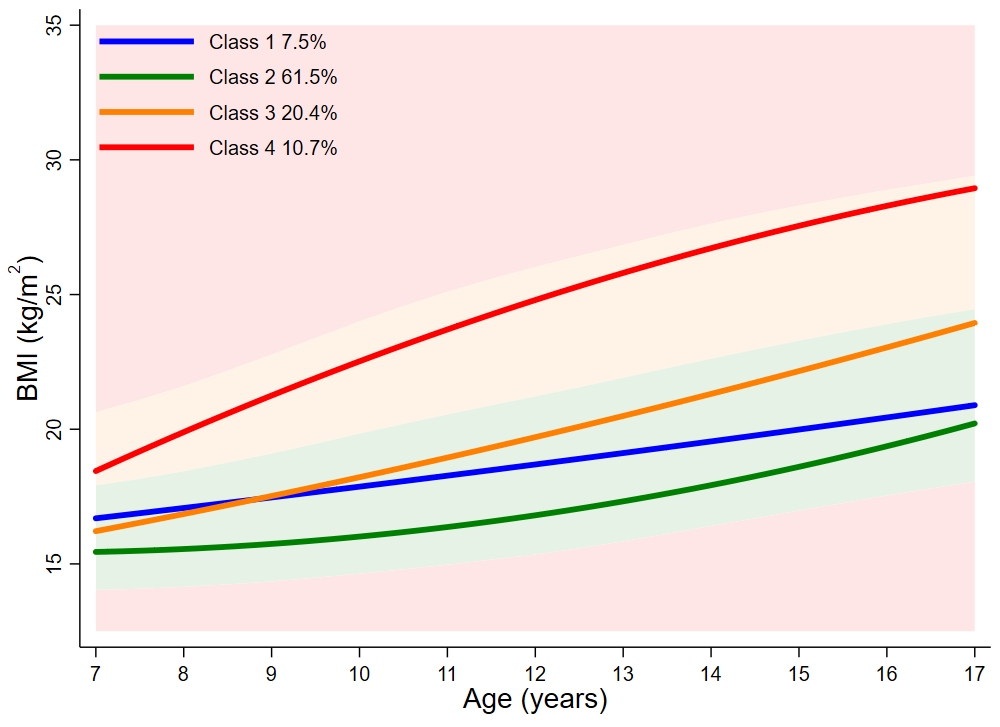


Supplementary Figure 1e. Final mixture model trajectories for a 5-class solution for boys


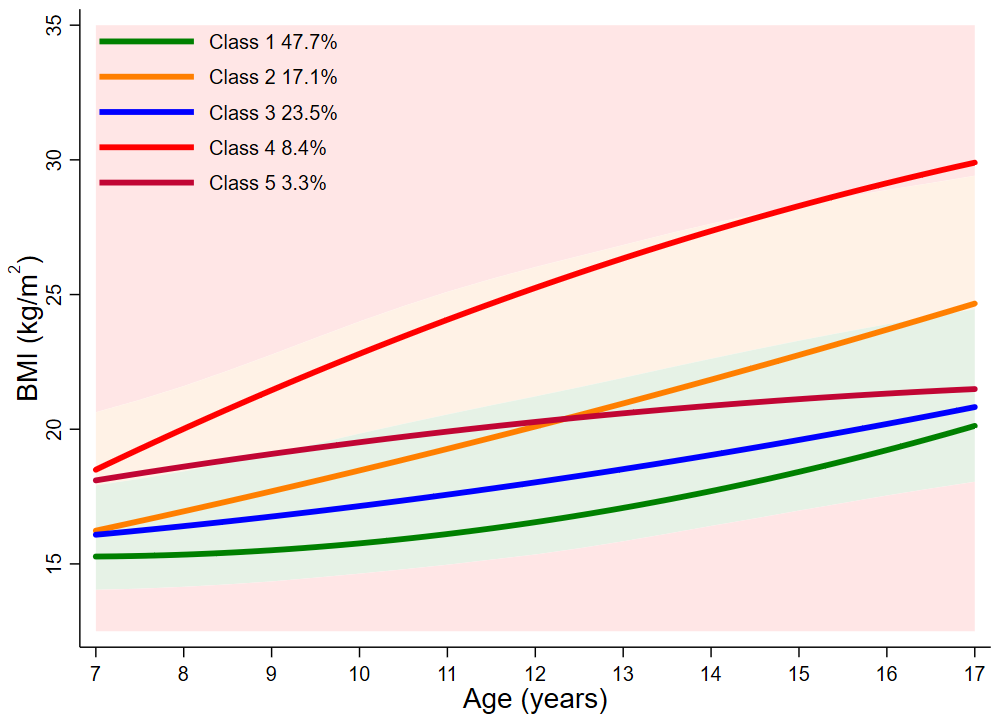


Supplementary Figure 1f. Final mixture model trajectories for a 6-class solution for boys


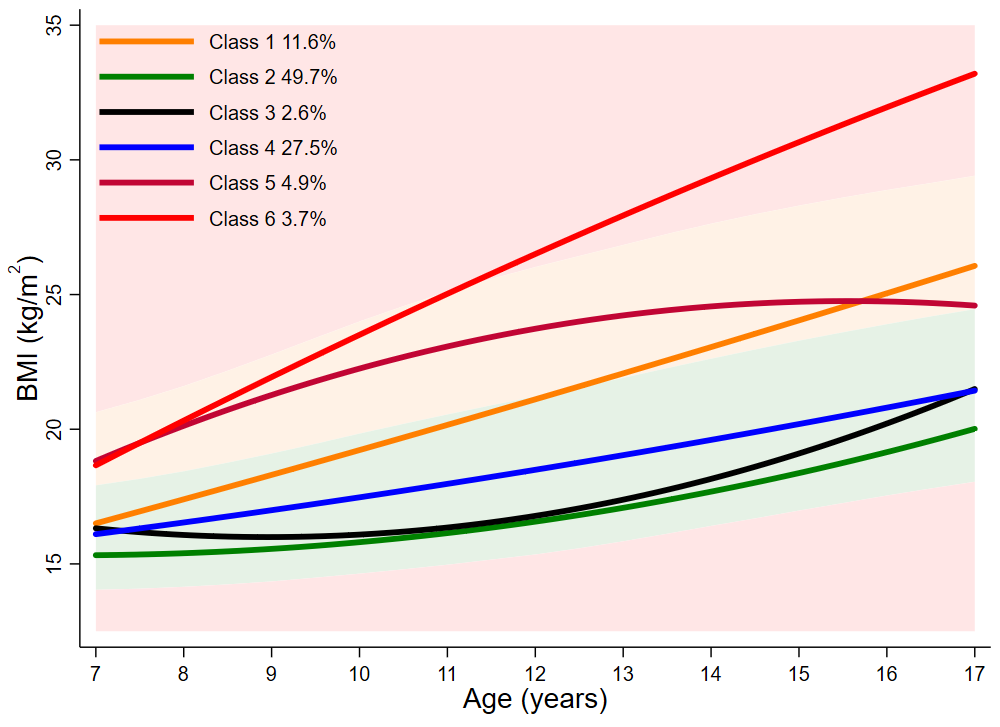


Supplementary Figure 2a. Final mixture model trajectories for a 1-class solution for girls


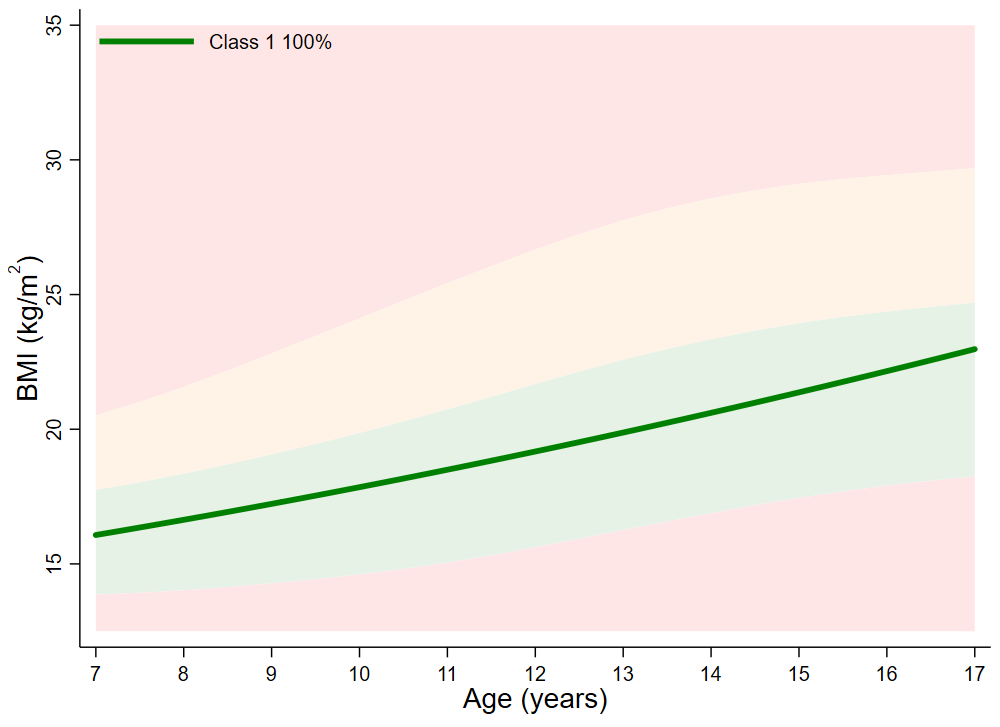


Supplementary Figure 2b. Final mixture model trajectories for a 2-class solution for girls


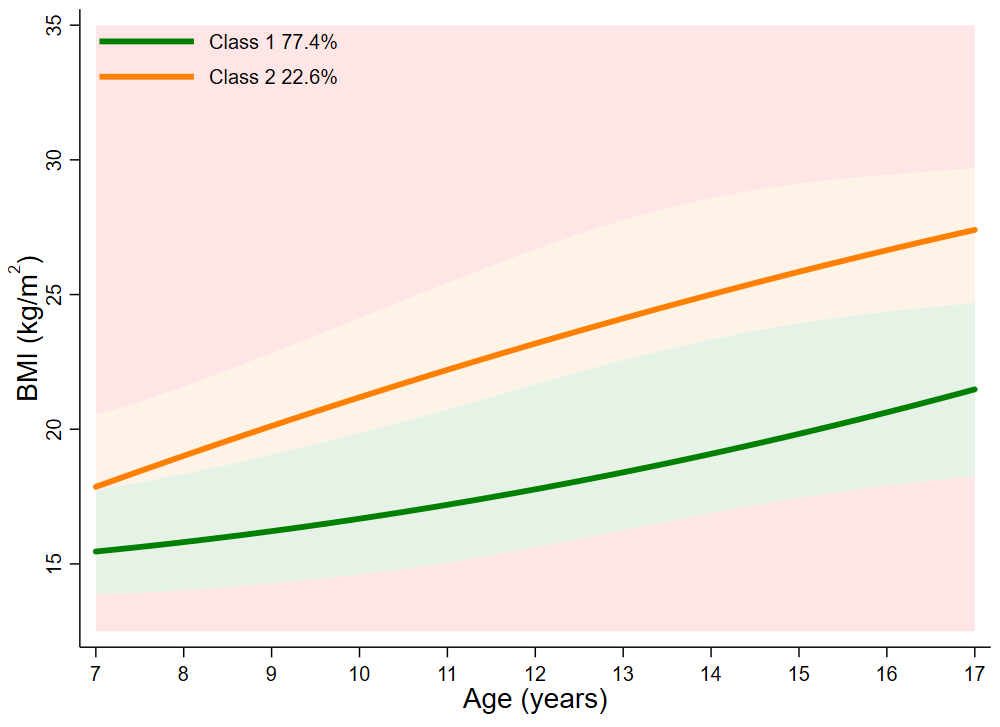


Supplementary Figure 2c. Final mixture model trajectories for a 3-class solution for girls


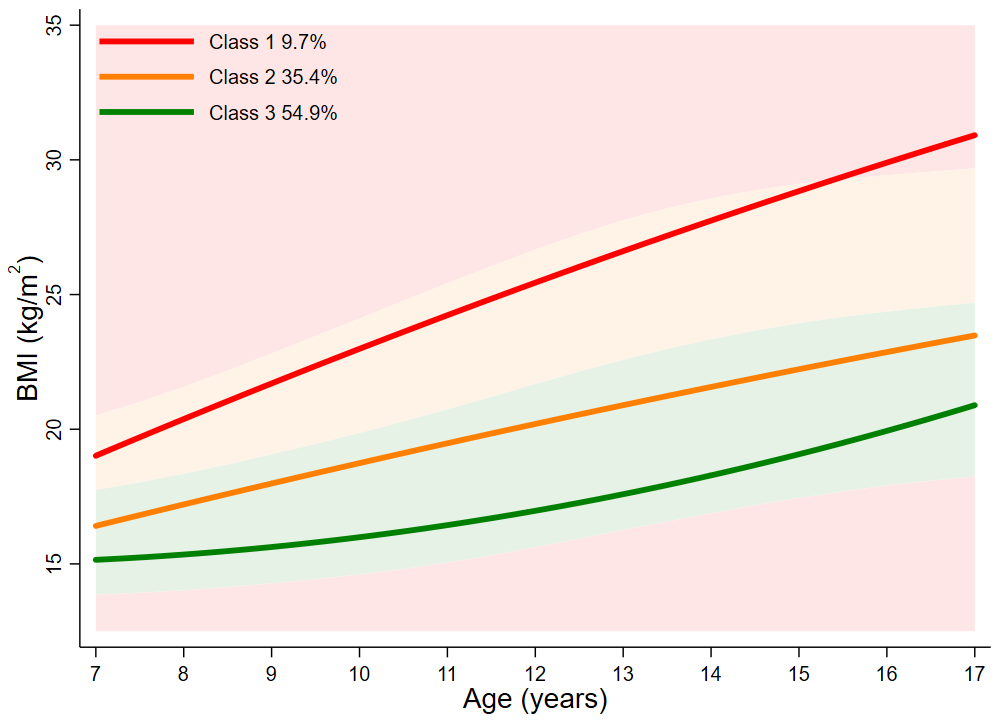


Supplementary Figure 2d. Final mixture model trajectories for a 4-class solution for girls


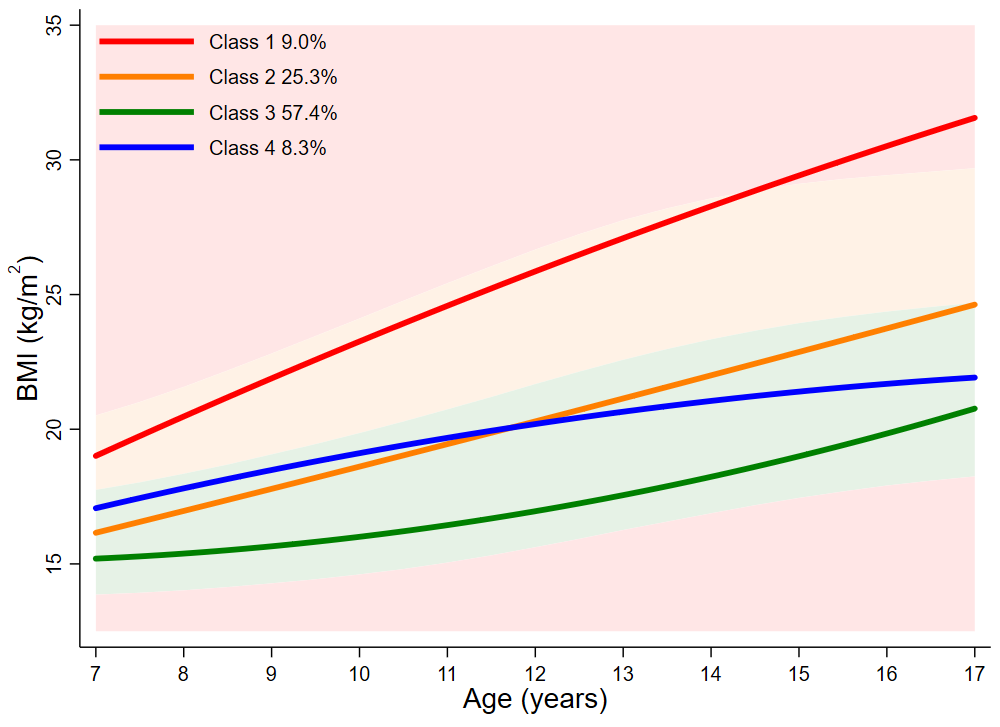


Supplementary Figure 2e. Final mixture model trajectories for a 5-class solution for girls


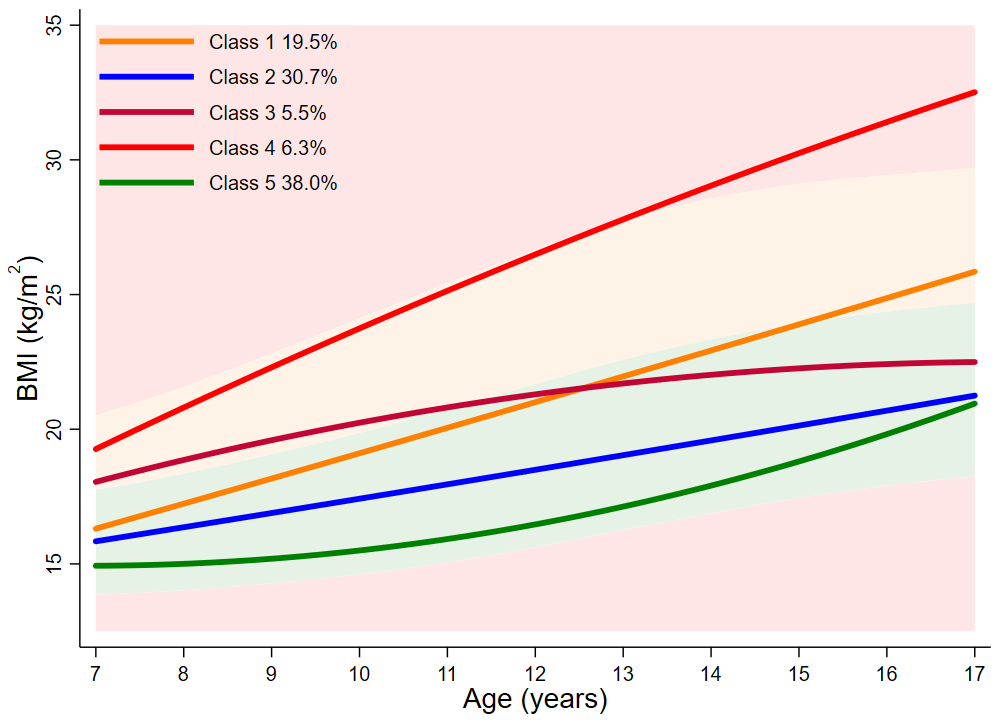


Supplementary Figure 2f. Final mixture model trajectories for a 6-class solution for girls


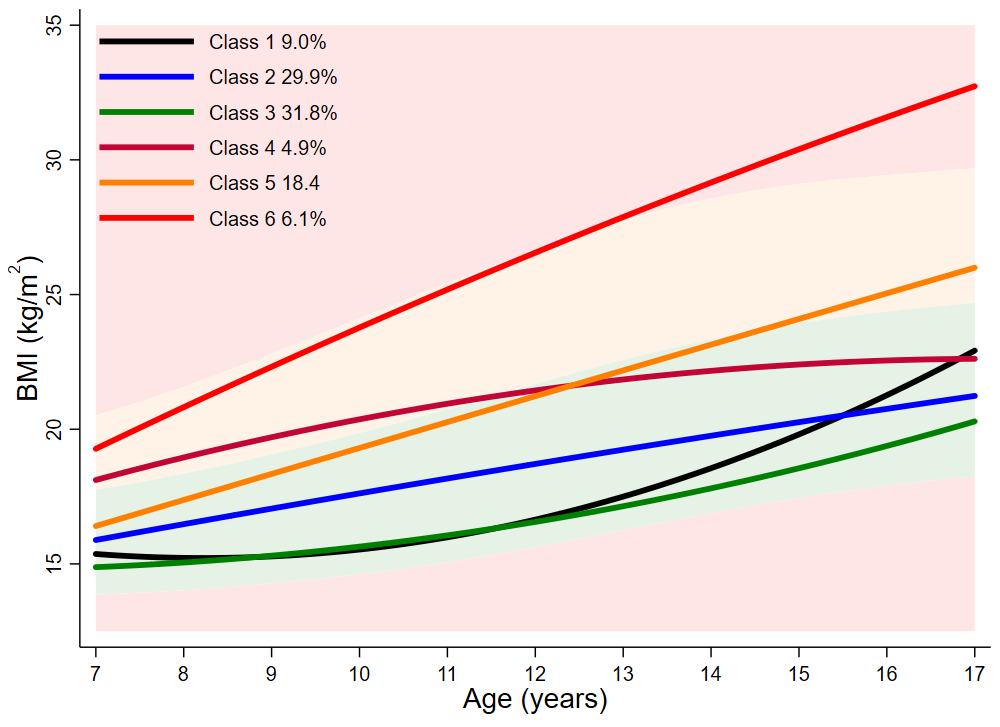


Supplementary Figure 3a. Average fitted trajectory (with 95% CIs) and individual observed trajectories for Class 1 in the final mixture model for boys


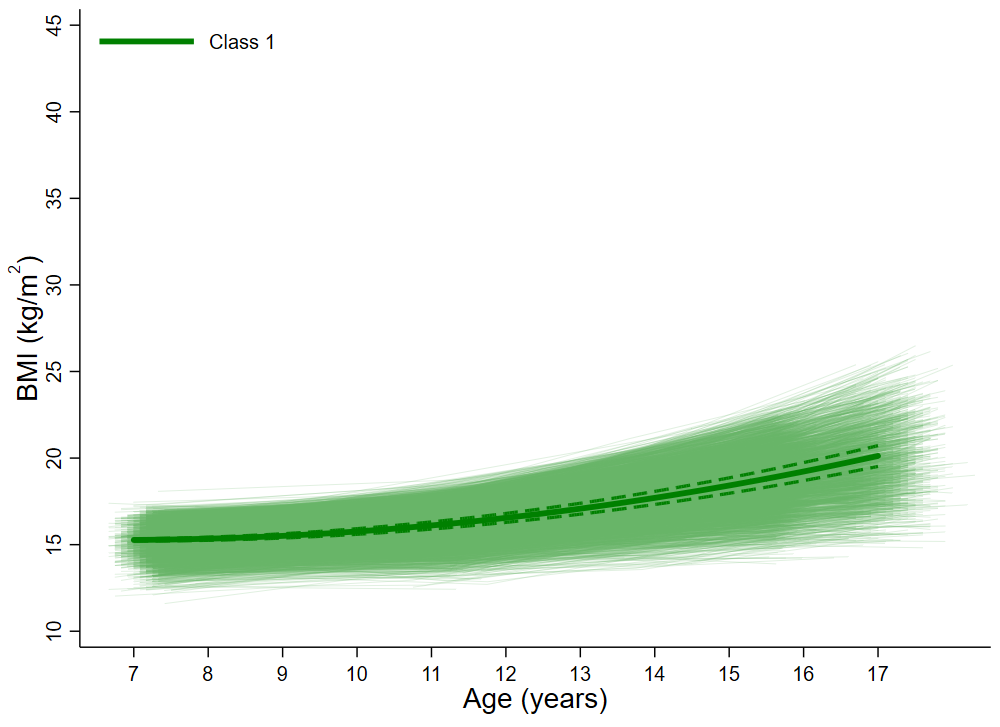


Supplementary Figure 3b. Average fitted trajectory (with 95% CIs) and individual observed trajectories for Class 2 in the final mixture model for boys


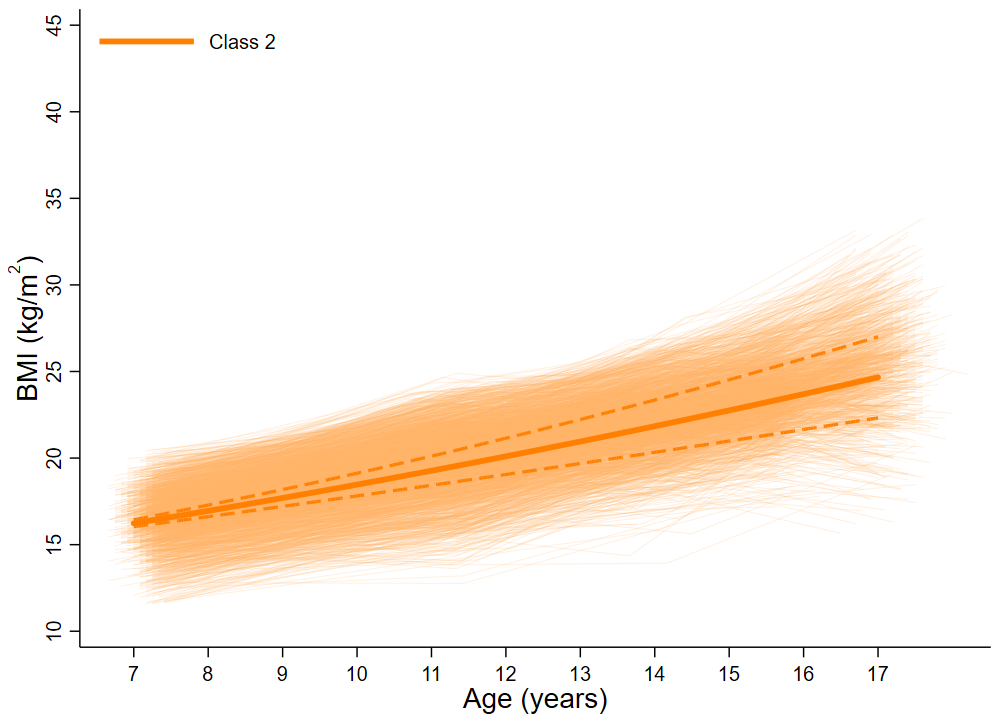


Supplementary Figure 3c. Average fitted trajectory (with 95% CIs) and individual observed trajectories for Class 3 in the final mixture model for boys


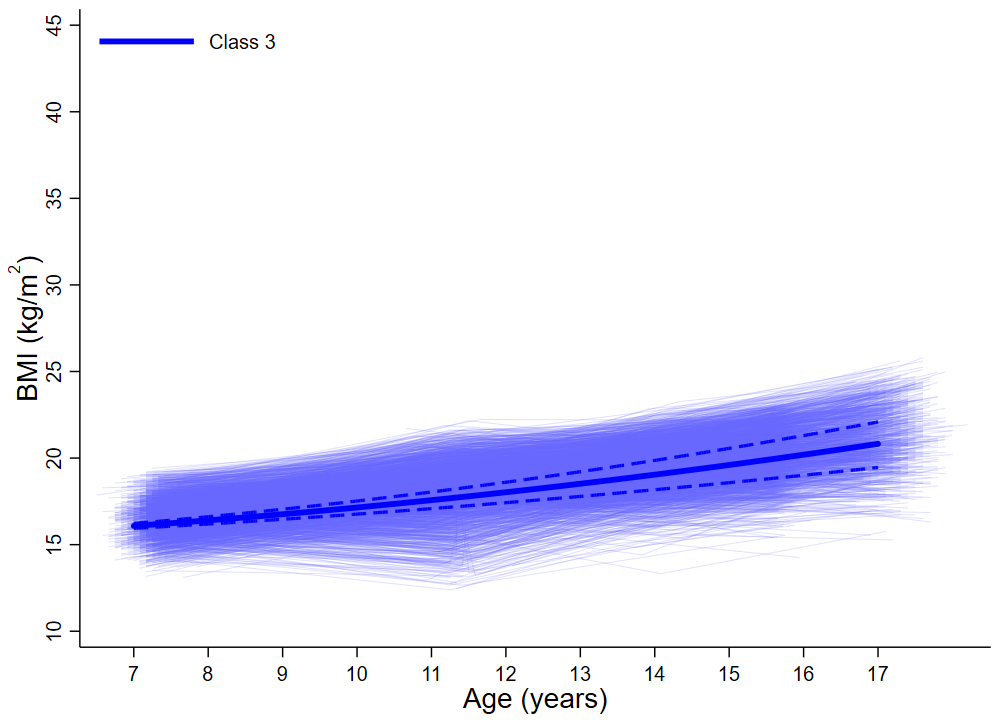


Supplementary Figure 3d. Average fitted trajectory (with 95% CIs) and individual observed trajectories for Class 4 in the final mixture model for boys


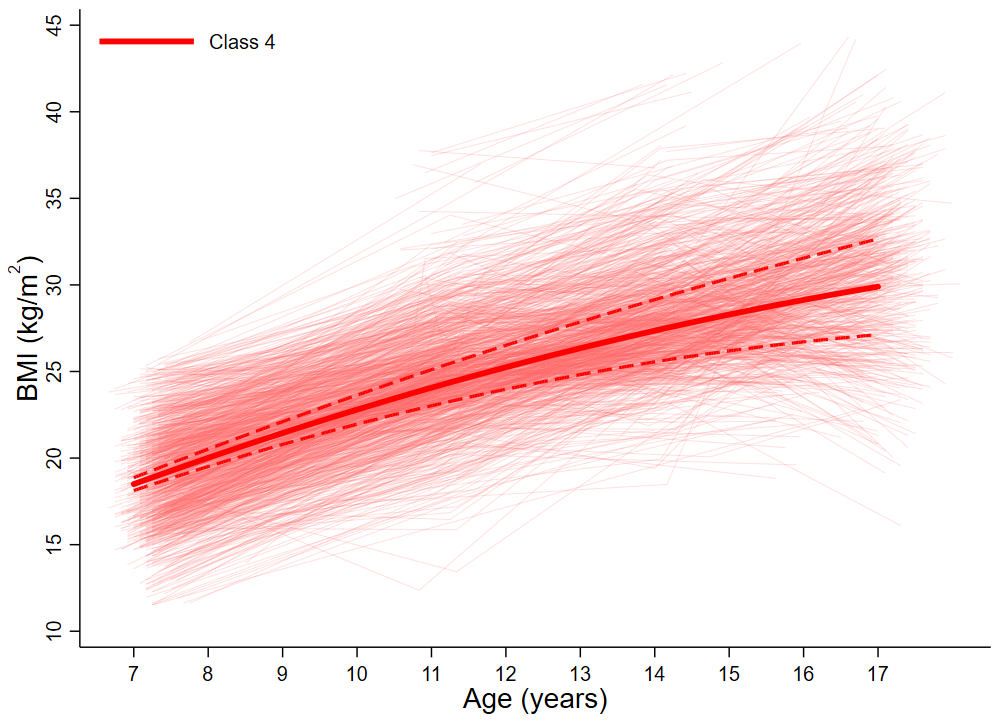


Supplementary Figure 3e. Average fitted trajectory (with 95% CIs) and individual observed trajectories for Class 5 in the final mixture model for boys


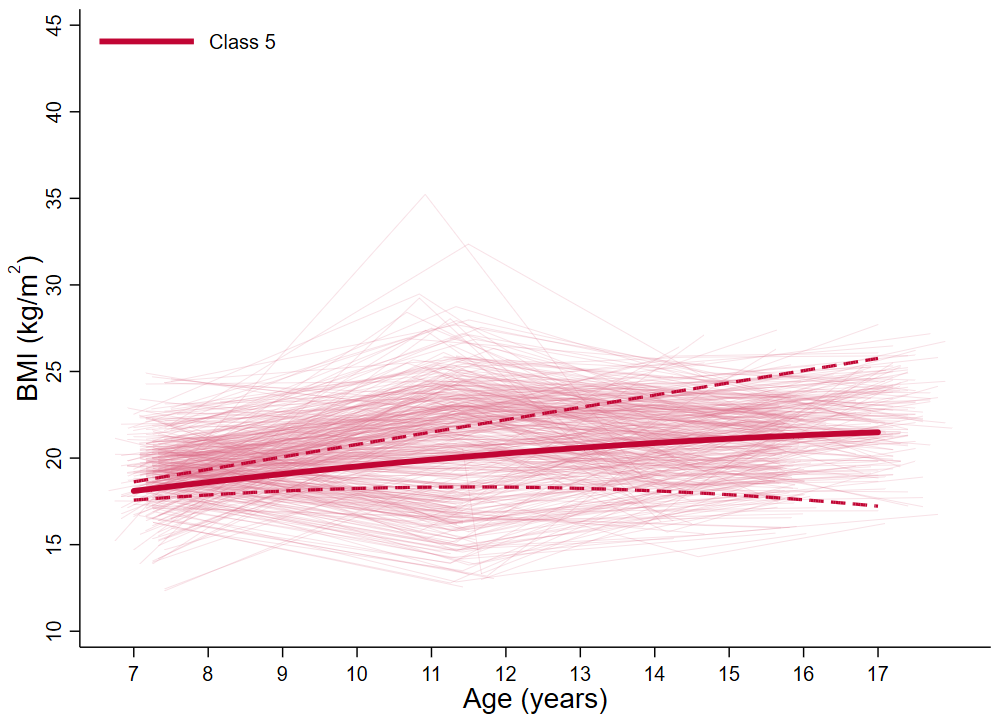


Supplementary Figure 4a. Average fitted trajectory (with 95% CIs) and individual observed trajectories for Class 1 in the final mixture model for girls


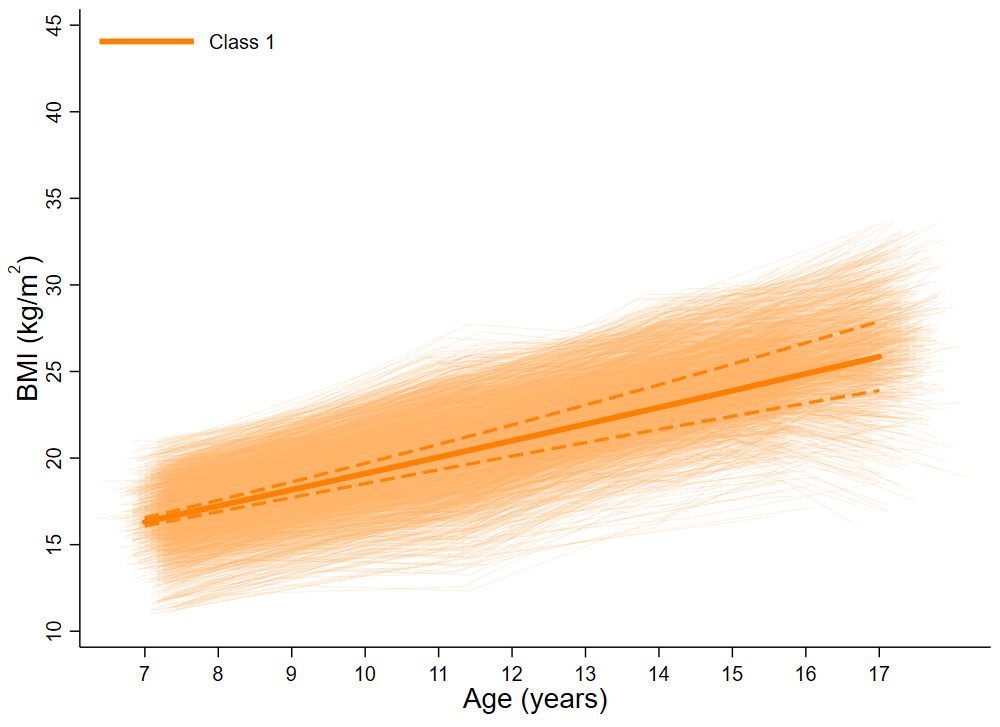


Supplementary Figure 4b. Average fitted trajectory (with 95% CIs) and individual observed trajectories for Class 2 in the final mixture model for girls


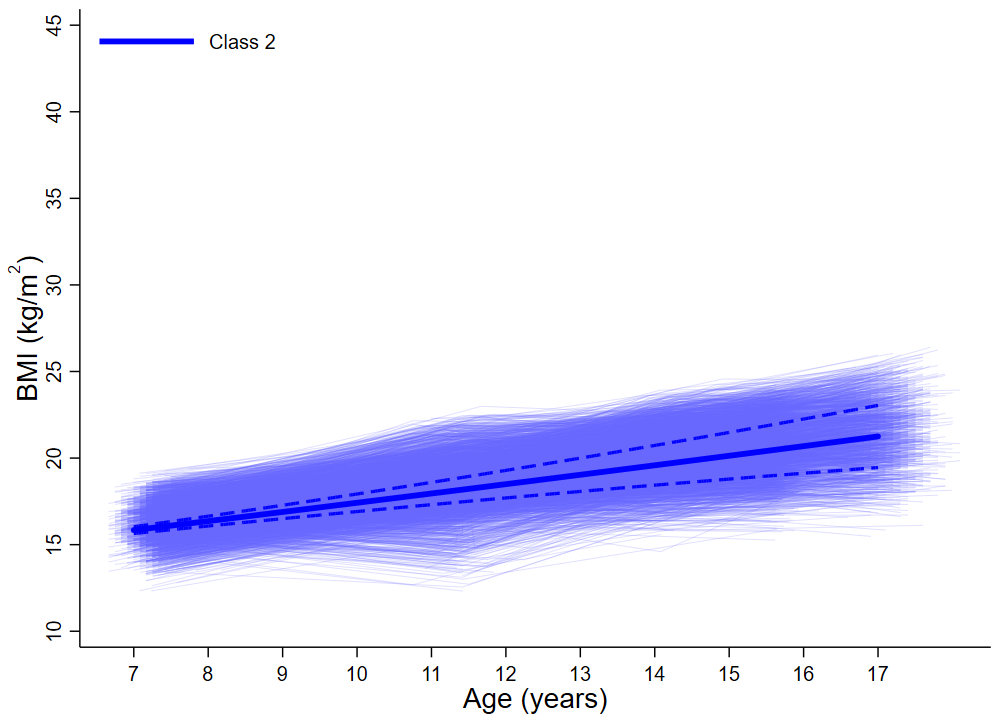


Supplementary Figure 4c. Average fitted trajectory (with 95% CIs) and individual observed trajectories for Class 3 in the final mixture model for girls


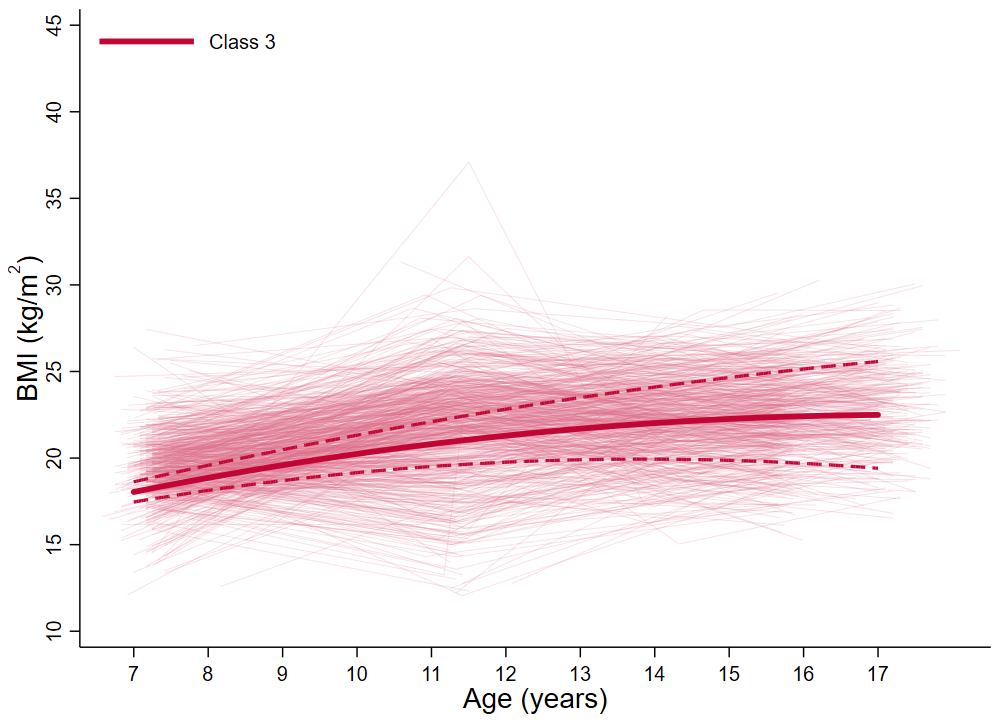


Supplementary Figure 4d. Average fitted trajectory (with 95% CIs) and individual observed trajectories for Class 4 in the final mixture model for girls


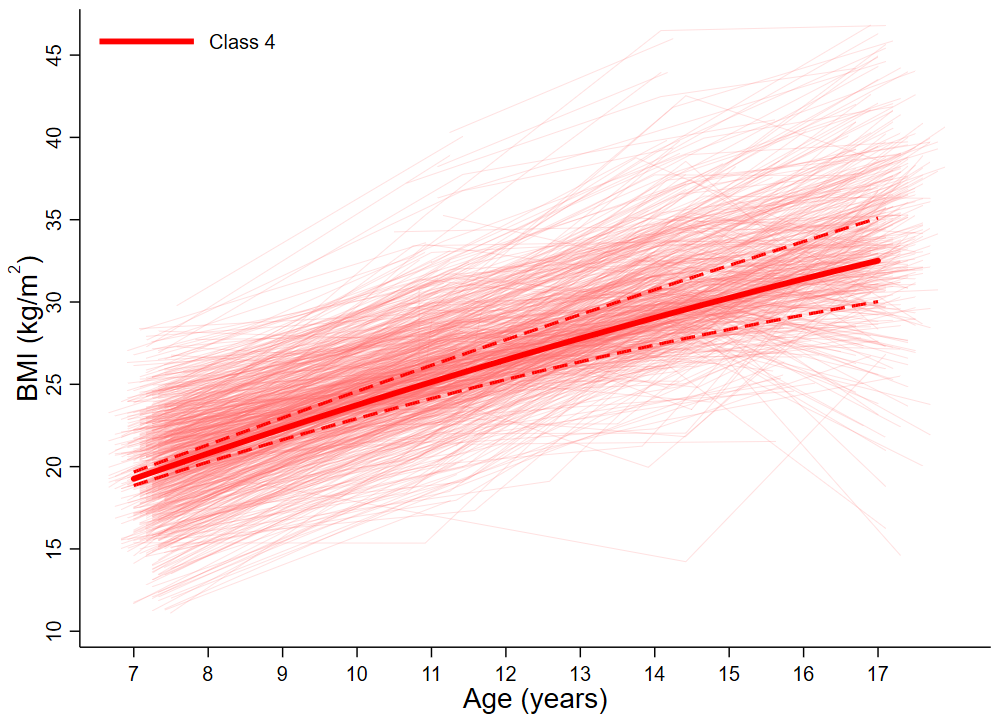


Supplementary Figure 4e. Average fitted trajectory (with 95% CIs) and individual observed trajectories for Class 5 in the final mixture model for girls


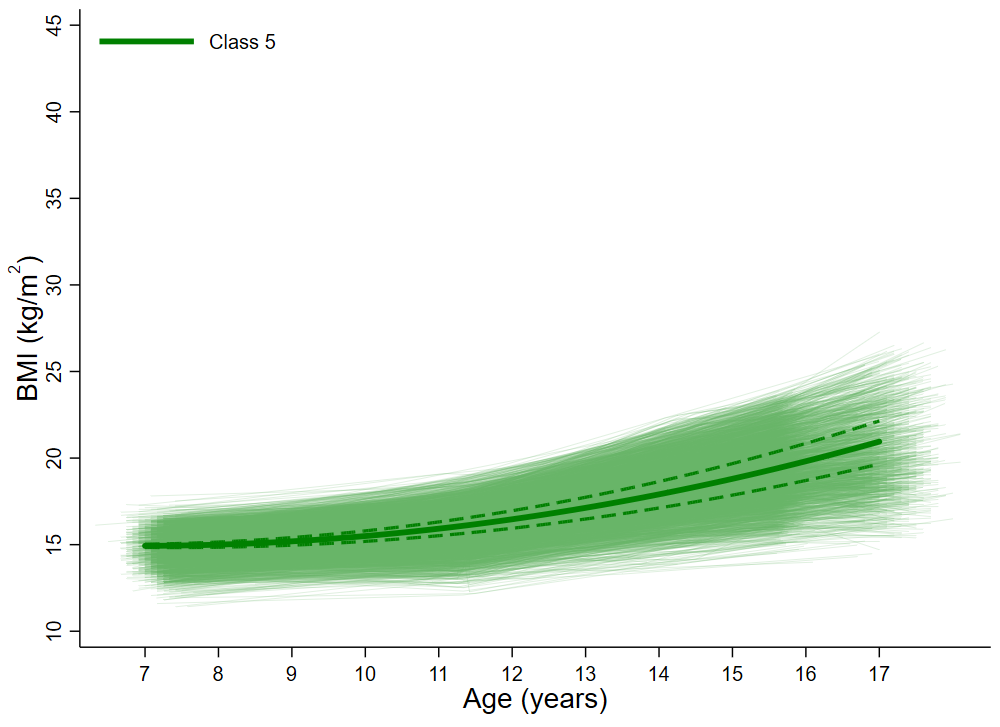


Supplementary Figure 5a. Distribution of posterior probabilities for assigned class membership for Class 1 in the final mixture model for boys


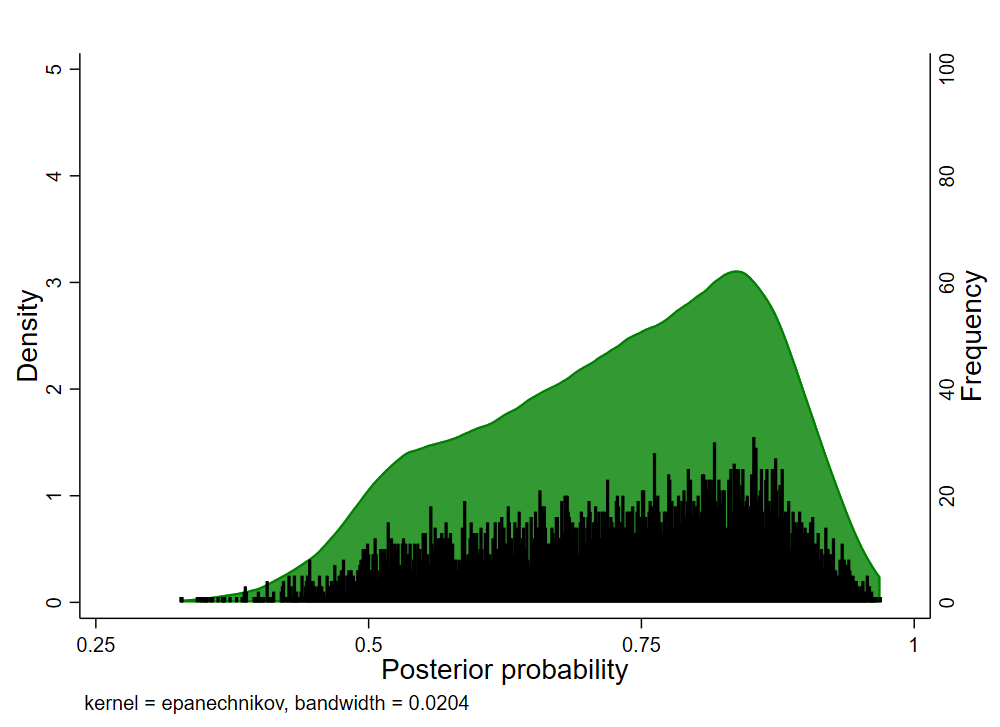


Supplementary Figure 5b. Distribution of posterior probabilities for assigned class membership for Class 2 in the final mixture model for boys


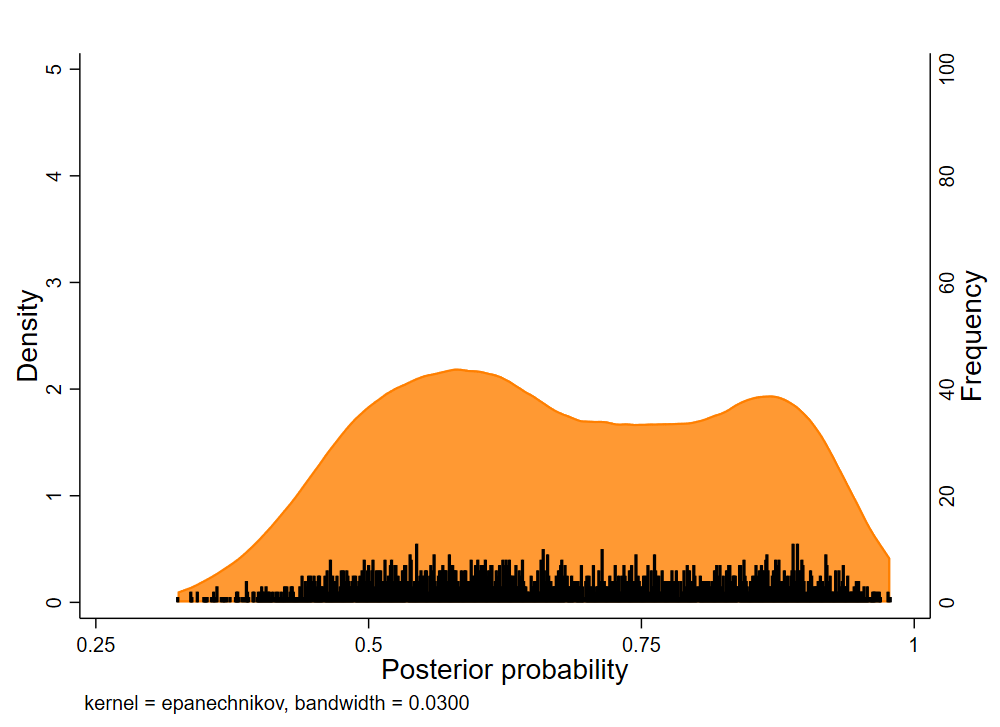


Supplementary Figure 5c. Distribution of posterior probabilities for assigned class membership for Class 3 in the final mixture model for boys


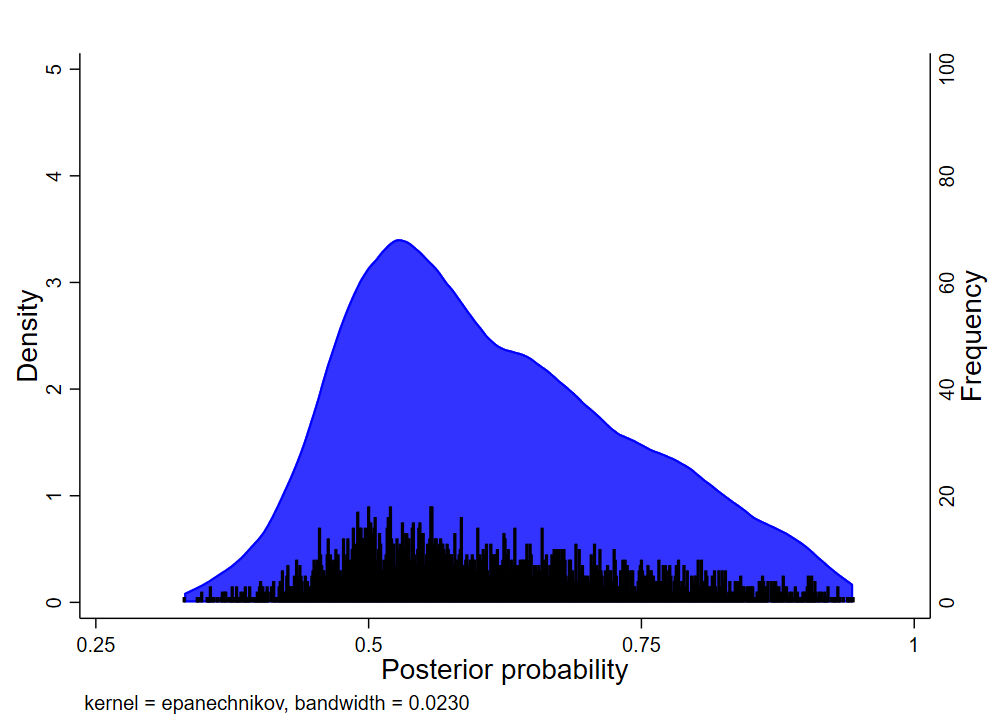


Supplementary Figure 5d. Distribution of posterior probabilities for assigned class membership for Class 4 in the final mixture model for boys


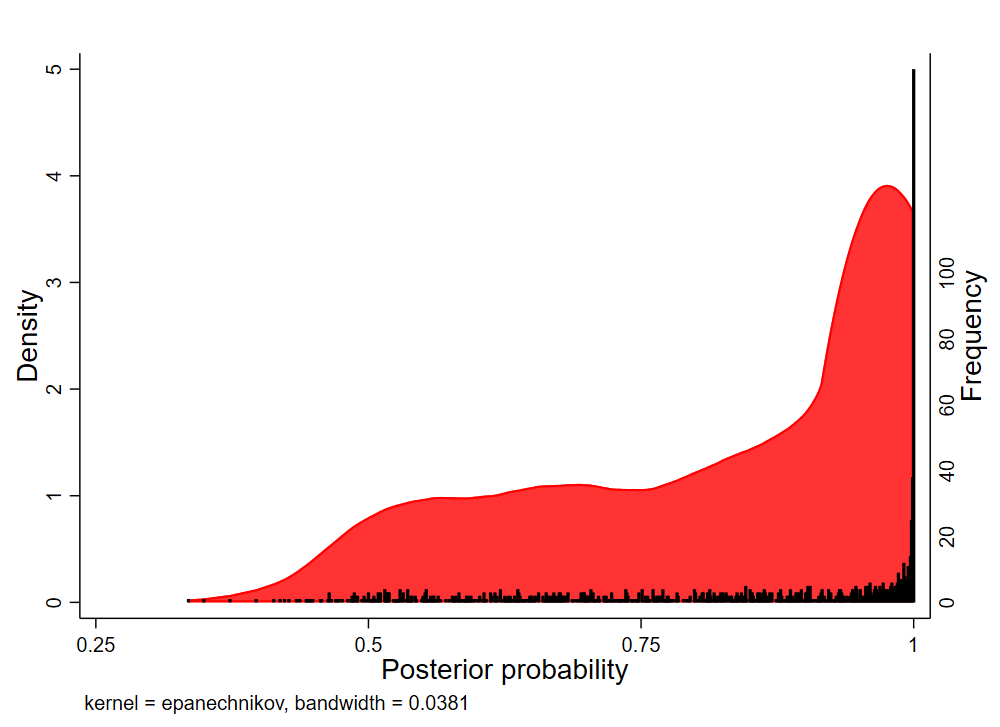


Supplementary Figure 5e. Distribution of posterior probabilities for assigned class membership for Class 5 in the final mixture model for boys


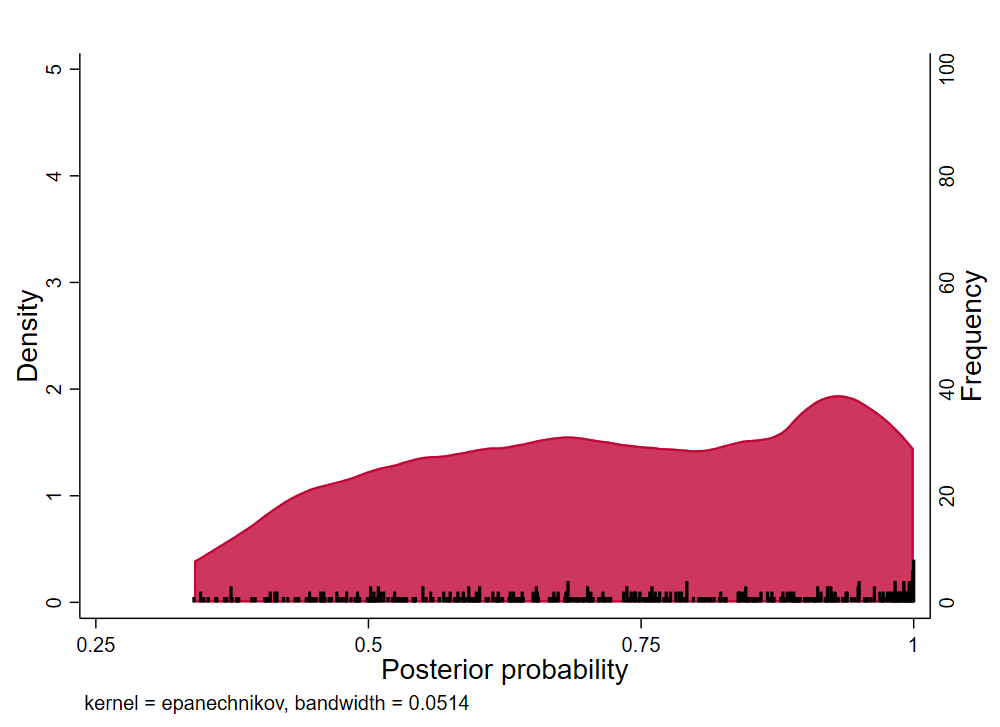


Supplementary Figure 6a. Distribution of posterior probabilities for assigned class membership for Class 1 in the final mixture model for girls


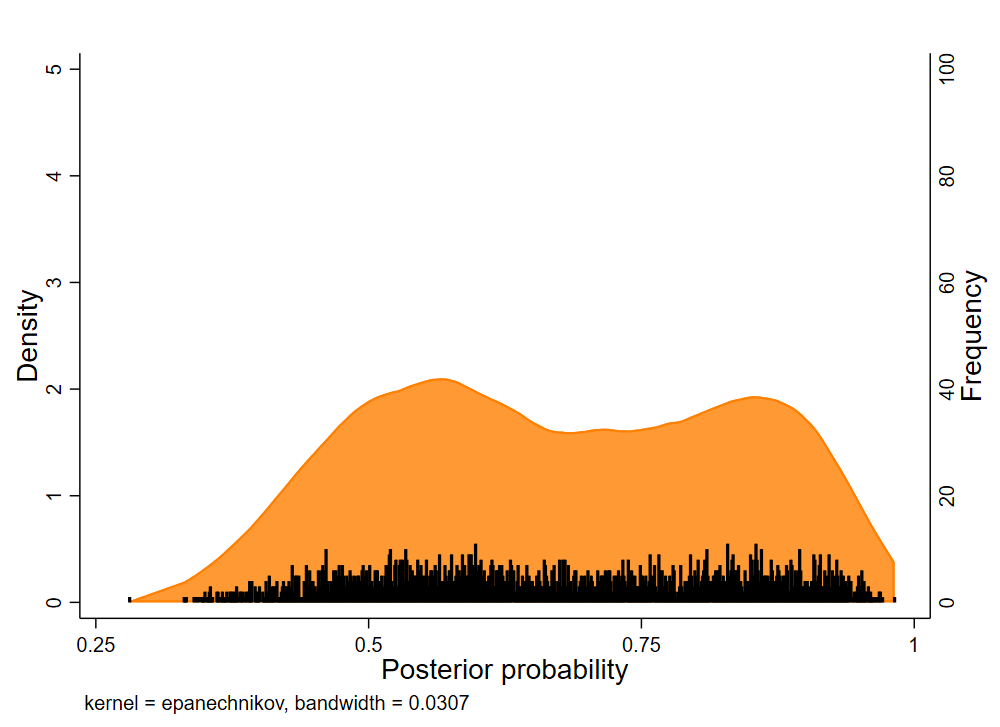


Supplementary Figure 6b. Distribution of posterior probabilities for assigned class membership for Class 2 in the final mixture model for girls


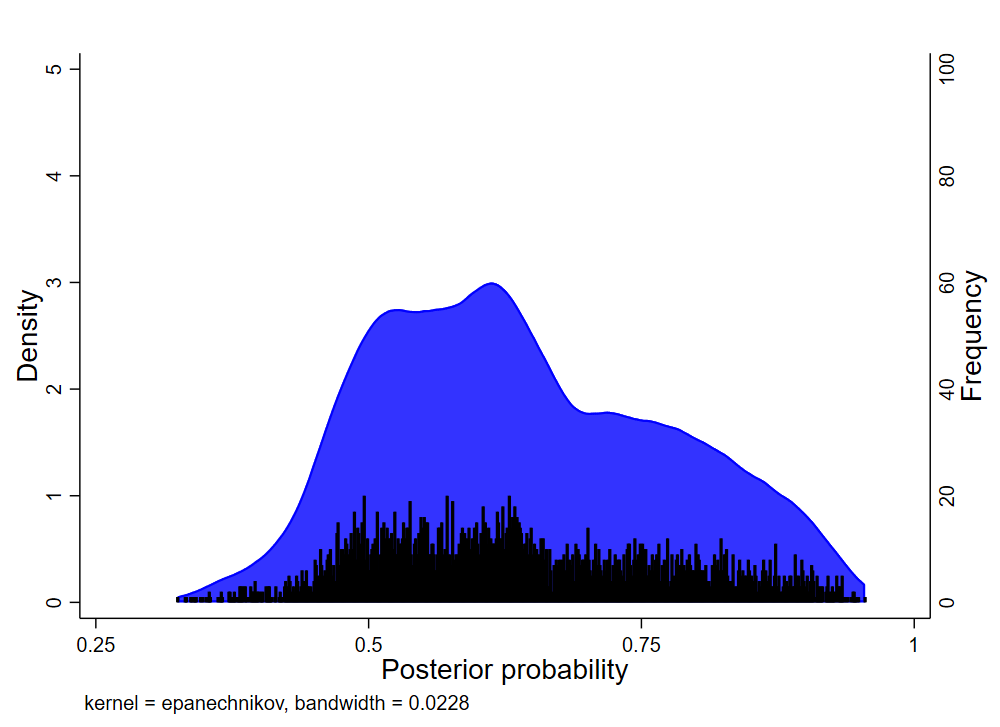


Supplementary Figure 6c. Distribution of posterior probabilities for assigned class membership for Class 3 in the final mixture model for girls


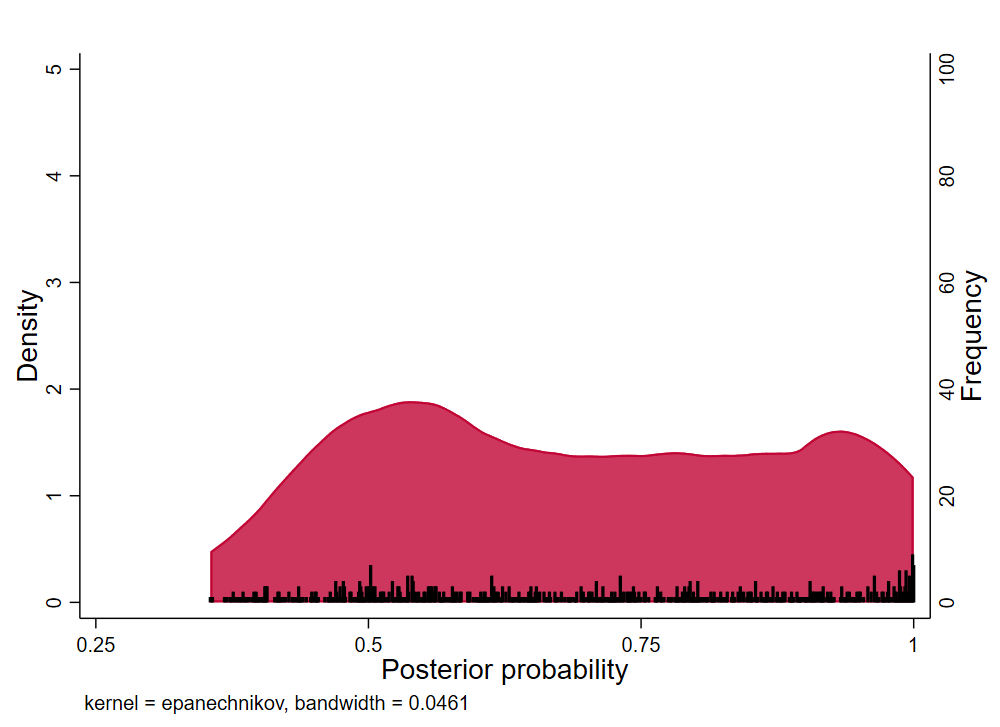


Supplementary Figure 6d. Distribution of posterior probabilities for assigned class membership for Class 4 in the final mixture model for girls


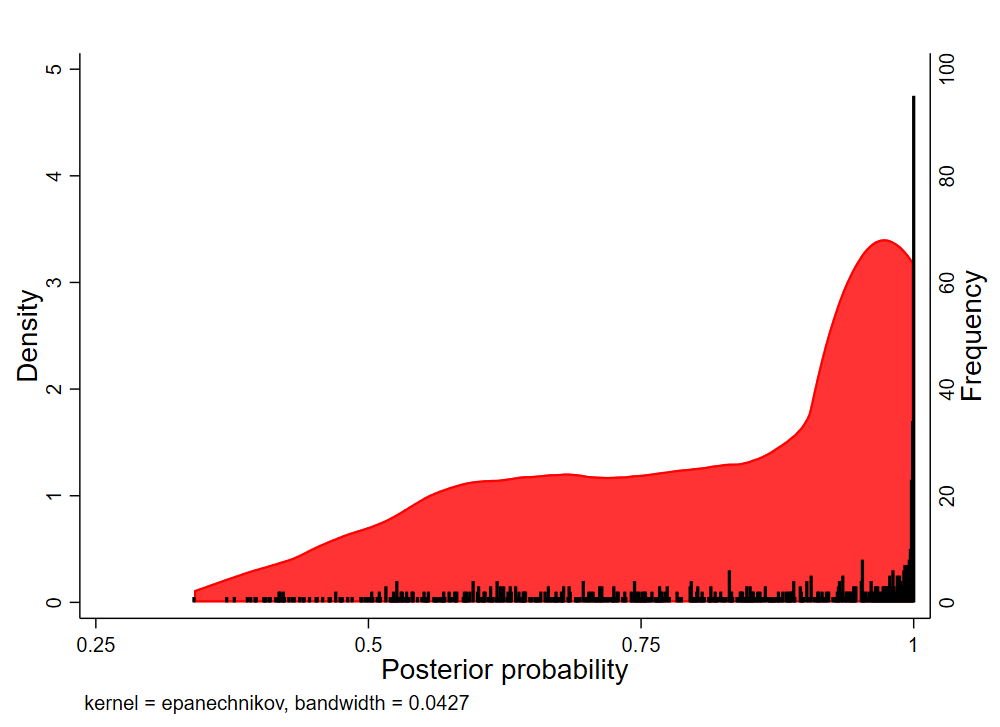


Supplementary Figure 6e. Distribution of posterior probabilities for assigned class membership for Class 5 in the final mixture model for girls


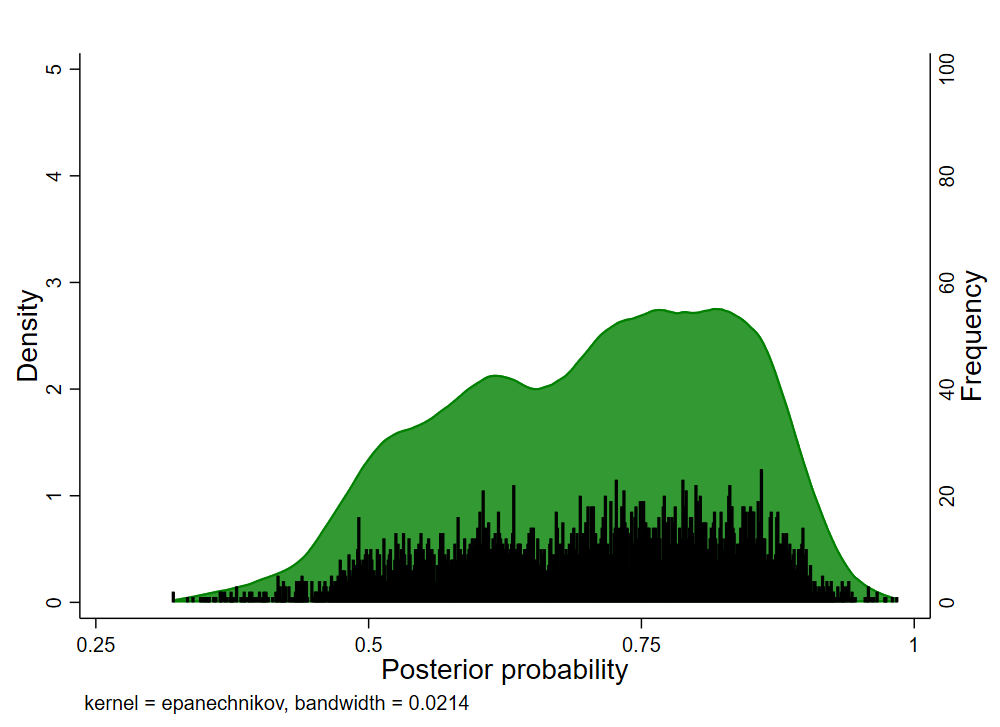

Supplement: SUPPLEMENTAL MATERIAL [file EMS156525-supplement-SUPPLEMENTAL_MATERIAL.docx]
